# Managing Fractured Ceramic Restorations: Current Evidence and Best Practices—A Scoping Review

**DOI:** 10.1111/jerd.70055

**Published:** 2025-12-31

**Authors:** Gabriela Almeida, Joana A. Marques, Markus B. Blatz, Rui I. Falacho

**Affiliations:** ^1^ Institute of Implantology and Prosthodontics, Faculty of Medicine, University of Coimbra Coimbra Portugal; ^2^ Institute of Paediatric and Preventive Dentistry, Faculty of Medicine, University of Coimbra Coimbra Portugal; ^3^ Center for Innovation and Research in Oral Sciences (CIROS) Faculty of Medicine, University of Coimbra Coimbra Portugal; ^4^ Department of Preventive and Restorative Sciences University of Pennsylvania School of Dental Medicine Philadelphia Pennsylvania USA; ^5^ ADEMA University School, University of the Balearic Islands (UIB) Palma Spain

**Keywords:** ceramic fracture, ceramics, intraoral repair, restorative dentistry, surface treatments

## Abstract

**Objective:**

This scoping review aims to assess the effect of different protocols on the outcome of ceramic restoration repairs.

**Materials and Methods:**

An electronic search was conducted in three databases (PubMed, Cochrane Library, and Embase), up to July 31st 2025, with previously identified MeSH terms.

**Results:**

Out of the 2423 records, title and abstract screening resulted in the exclusion of 2136 and 136 studies, respectively, while full‐text analysis excluded another 37 articles. In addition, 10 records were not included, as full texts could not be obtained after requesting the authors. Twenty‐two cross‐references were added. Thus, 126 studies were included in this review. It is important to emphasize the methodological heterogeneity among studies. Bond strength was the most frequently evaluated outcome, followed by ultrastructural analysis, chemical analysis, color evaluation and mechanical properties.

**Conclusions:**

In ceramic repair procedures, glass ceramics should be treated with a combination of hydrofluoric acid etching and silanization, while metal oxide ceramics demonstrate superior outcomes when air abrasion and an MDP‐containing primer are employed.

## 1 Introduction

Ceramic restorations have gained widespread use owing to their favorable esthetic and mechanical properties, enabling a broad range of clinical applications [1]. Ceramics are typically classified as either glass or metal oxide ceramics, with the former considered acid‐sensitive and the latter acid‐resistant [2]. Metal oxide ceramics are further categorized into alumina and zirconia, with zirconia emerging as the most employed material in recent years due to their biocompatibility and superior mechanical performance [3, 4]. Glass ceramics, including feldspathic, leucite‐reinforced and lithium disilicate ceramics, are composed of a silica matrix and recognized for their satisfactory optical characteristics [3, 4].

Despite the high success rates associated with these materials, chipping and fractures have been reported [4, 5]. This can be caused by either patient‐related factors, such as parafunctional habits and occlusal patterns, or by restoration‐related features, including design, an inadequate bonding protocol, occlusal adjustments, or the presence of internal defects [4].

Ceramic repair protocols have been introduced taking into account esthetic, functional and biological considerations [6]. In most cases, this procedure allows for a more efficient and conservative approach compared to restoration replacement, as no additional tooth preparation is required and structural integrity and pulp vitality are not compromised [1, 4, 7, 8, 9, 10]. Numerous protocols, either micromechanical or chemical, have been proposed to establish an adequate repair procedure according to the type of restoration and type of fracture [1, 7, 8, 11]. Therefore, structural analyses of treated ceramics and bond strength evaluation of repaired ceramic restorations have been conducted over the years, with the literature presenting heterogeneous results [2].

Literature lacks an adequate systematic and comprehensive review of the different available ceramic repair protocols, considering not only bond strength outcomes, but also surface topography alterations, chemical modifications, esthetic results over time, and other success outcome measures. To date, no comprehensive review has simultaneously addressed these diverse outcomes across all ceramic material types. Given the wide variety of study designs and the need to map the existing evidence, this scoping review aims to assess the effect of different protocols on the outcomes of ceramic restoration repairs.

## 2 Methodology

This review was reported according to the recommendations of the Preferred Reporting Items for Systematic Reviews and Meta‐Analyses statement, extension for Scoping Reviews (PRISMA‐ScR; Data S1), along with the methodological guidelines outlined by Prott et al., to ensure rigorous reporting [12, 13].

### 2.1 Eligibility Criteria

Inclusion criteria comprised studies written in English that assessed different strategies for ceramic restoration repair. Exclusion criteria included publications that did not mention restoration repair, addressed hybrid or metal ceramics, or employed repair materials other than ceramics or composite resins. In addition, case reports, technique description articles, and papers in which the repair protocol was not fully disclosed were also excluded.

### 2.2 Information Sources and Search Strategy

A literature search was performed up to July 31, 2025, on PubMed (via Medline), Cochrane Library and Embase. In order to define the search key (Table 1), the following MeSH/Emtree terms were identified: “ceramics,” “dental restoration repair,” “dental bonding,” “tensile strength,” “shear strength,” “flexural strength,” “elastic modulus,” and “survival rate.” A time restriction was applied, with studies up to 2004 not to be considered.

**TABLE 1:** Search strategy.

Search terms	#1	“ceramics” OR “oxide‐ceramic” OR “oxide ceramic” OR “glass–ceramic” OR “glass ceramic” OR “alumina” OR “zirconia” OR “lithium disilicate” OR “leucite” OR “feldspathic”
	#2	“repair” OR “ceramic repair” OR “reparation” OR “rebonding”
	#3	“dental bonding” OR “bond strength” OR “tensile strength” OR “shear strength” OR “mechanical properties” OR “biomechanics” OR “flexural strength” OR “fracture resistance” OR “elastic modulus” OR “elasticity modulus” OR “microhardness” OR “survival rate” OR “color stability” OR “colour stability” OR “aesthetic integration” OR “esthetic integration” OR “methods” OR “technique”
MeSH terms/Emtree terms	#4	“ceramics”
	#5	“dental restoration repair”
	#6	“dental bonding” OR “tensile strength” OR “shear strength” OR “flexural strength” OR “elastic modulus” OR “survival rate” OR “methods”
Search key	(#1 OR #4) AND (#2 OR #5) AND (#3 OR #6)	

### 2.3 Selection of Sources of Evidence

The search results from each database were transferred to Zotero reference manager and duplicate identification and removal were conducted. Publications were firstly screened based on title and abstract according to the eligibility criteria, by two independent reviewers. The selected articles were further submitted to a full‐text analysis. In case of disagreement, a third researcher was involved in the eligibility process. To identify cross‐references, all included articles were screened to assess potentially relevant studies within the reference lists.

### 2.4 Data Extraction

Data extraction from the included studies comprised type of study, sample size, restorative and repair material, control and experimental groups, evaluated outcome, aging, and main findings.

## 3 Results

**FIGURE 1:**
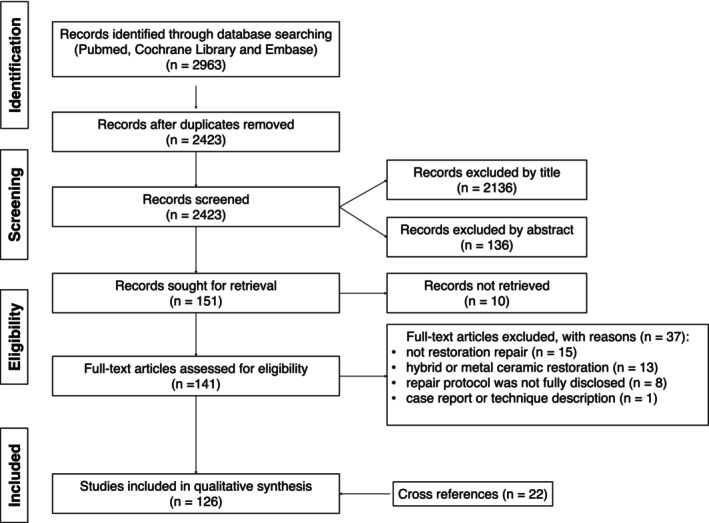
Flowchart of the study selection.

The flowchart of the study selection process is presented in Figure 1. The literature search identified 2963 publications, with 2423 remaining after duplicate removal. Title and abstract screening resulted in the exclusion of 2136 and 136 studies, respectively. Subsequently, full‐text analysis excluded 37 articles, while the full text of 10 records could not be retrieved after requesting the authors. In addition, 22 cross‐references were added and a total of 126 studies were included in this review. Methodological data and main results are summarized in Table 2.

**TABLE 2:** Data extraction of the included studies.

Authors (year)	Type of study	*n*	Restorative material	Control group	Experimental groups	Common procedure	Repair material	Outcome	Aging	Main findings
Abdulla and Hasan (2022)	In vitro	*n* = 5	Zirconia (DD BioZ Wiso), feldspathic (VITA VM 9), and zirconia with feldspathic ceramic	No surface treatment	Er,Cr:YSGG laser (2780 nm, 300 mJ, 3 W, 10 Hz, 140 μs, at 6 mm for 10 mm) and air abrasion with 50 μm aluminum oxide (10 mm, 90°, 5 s, 80 psi); Cimara Repair System and Ceramic Repair N	—	Composite resin (Tetric N‐Ceram and Arabesk Top)	Bond strength (shear)	Yes	Air abrasion was associated with superior bond strength values in comparison to laser irradiation
Akyil et al. (2010)	In vitro	*n* = 13	Feldspathic ceramic (Vita VMK)	No surface treatment	9.5% Hydrofluoric acid (2 min), Er:YAG laser (2940 nm, 1 min, 3 W, 300 mJ, 10 Hz), Nd:YAG laser (1064 nm, 1 min, 1 W, 100 mJ, 10 Hz), Er:YAG laser + hydrofluoric acid, Nd:YAG laser + hydrofluoric acid	Cimara repair system	Composite resin (Arabesk Top)	Bond strength (shear) and surface topography analysis (SEM)	Yes	Hydrofluoric acid etching proved to be the most efficient surface treatment to enhance the bond strength between a repair composite resin and feldspathic ceramic
Al Deeb (2020)	In vitro	*n* = 10	Lithium disilicate (IPS e.max press) and zirconia (Noritake Alliance)	Diamond bur (30‐μm grit)	Er,Cr:YSGG laser (2.78 μm, 3.75 W, 15 Hz, 60 s), Nd:YAG laser (1064 nm, 2 mm, 60 s, 150 mJ, 10 Hz, 3 W), 9.6% hydrofluoric acid (60 s) + silane, air abrasion with 30 μm silica‐coated aluminum oxide (2.3 bar, 8 mm, 1 min) + silane	Adhesive resin (Heliobond)	Composite resin (Multicore flow)	Bond stregth (shear)	Yes	For lithium disilicate, the use of hydrofluoric acid with silanization appears to be the gold standard. For zirconia, the combination of air abrasion and silane rendered the highest bond strength values
Al‐Askary et al. (2024)	In vitro	*n* = 7	Zirconia (H. C. Starck) and lithium disilicate (IPS E‐Max CAD)	—	9.5% Hydrofluoric acid (20 s), air abrasion with 50 μm aluminum oxide (20 s, 10 mm, 3 bar, 90°), diamond bur, Er,Cr:YSGG laser (2780 nm, 3 W, 10 Hz, 200 μs), diamond bur + 9.5% hydrofluoric acid (20 s), air abrasion with 50 μm aluminum oxide (20 s, 10 mm, 3 bar, 90°) + 9.5% hydrofluoric acid (20 s), Er,Cr:YSGG laser (2780 nm, 3 W, 10 Hz, 200 μs) + 9.5% hydrofluoric acid (20 s); G‐Premio Bond universal adhesive and intraoral repair kit	—	Composite resin (Filtek Z250)	Bond strength (shear)	No	The association of air abrasion and hydrofluoric acid led to the highest bond strength values
Al‐Hmadi et al. (2022)	In vitro	*n* = 10	Zirconia (H.C. Starck)	—	Bisco repair system (9.5% hydrofluoric acid + silane + bond), Clearfil repair system (40% phosphoric acid + Clearfil SE Bond Primer + Clearfil‐SE Bond + Clearfil Porcelain Bond Activator), Ivoclar ceramic repair system (Monobond Plus + Heliobond), Single Bond, Ultradent repair system (9% hydrofluoric acid + Ultradent silane, Peak Universal bond)	Air abrasion with 50 μm aluminum oxide (10s, 0.3 MPa, 10 mm)	Composite resin (Filtek Z250)	Bond strength (shear)	Yes	The repair of zirconia can be successfully achieved with Ivoclar ceramic repair system, Clearfil repair system and Bisco repair systems
Al‐Thagafi et al. (2016)	In vitro	NR	Lithium disilicate ceramic (IPS e.max) and zirconia‐reinforced lithium disilicate ceramic (Vita Suprinity)	No surface treatment	5% Hydrofluoric acid (60 s) + silane (Monobond), 5% hydrofluoric acid (60 s) + silane (Monobond) + adhesive (Heliobond), air abrasion with silica‐coated aluminum oxide (10 mm, 2.8 bar, 15 s) + silane (Monobond)	—	Composite resin (Tetric EvoCeram)	Bond strength (microtensile) and surface topography analysis (SEM)	Yes	The different tested surfaces treatements influence the repair bond strength of lithium disilicate ceramics
Al‐Turki et al. (2020)	In vitro	*n* = 1	Feldspathic (Vitablocks), zirconia‐reinforced lithium disilicate ceramic (Vita Suprinity) and yttria‐stabilized zirconia (Vita YZ T)	No surface treatment	5% Hydrofluoric acid (90 s), air abrasion with 50 μm aluminum oxide (5 mm, 10 s) and air abrasion with 45 μm silica‐coated (10 mm, 2.8 bar, 20 s)	Silane + adhesive system (Tetric N‐Collection)	Composite resin (Tetric N‐Collection)	Bond strength (microtensile)	Yes	The repair of feldspathic was associated with better results when hydrofluoric acid was used. On the other hand, the repair of zirconia and zirconia‐reinforced lithium disilicate ceramic resulted in lower bond strength
Aladağ and Ayaz (2023)	In vitro	*n* = 7	Zirconia (Upcera ST), zirconia‐reinforced lithium disilicate ceramic (Vita Suprinity) and lithium aluminosilicate ceramic reinforced with lithium disilicate (Nice block)	No surface treatment	Er:YAG laser (2940 nm, 2 W, 200 mJ, 10 Hz, 75 μs), Nd:YAG laser (1064 nm, 2 W, 100 mJ, 20 Hz, 150 μs), 5% hydrofluoric acid (20 s), hydrofluoric acid + Er:YAG laser, hydrofluoric acid + Nd:YAG laser, air abrasion with 50 μm aluminum oxide (2 bar, 10 mm, 15 s)	Clearfl Ceramic Primer Plus + bond (single bond universal adhesive)	Composite resin (Estelite Sigma Quick)	Bond strength (shear)	Yes	Laser irradiation enhanced the bond strength for Y‐TZP, while its combination with hydrofluoric acid provided a higher bond strength for ZLS and LD‐LAS ceramics
Alnassar (2017)	In vitro	*n* = 10	Zirconia	No surface treatment	Primer with MDP, air abrasion with 50 μm aluminum oxide (2.8 bar, 15 s, 10 mm), air abrasion with 50 μm aluminum oxide + primer with MDP, air abrasion with 30 μm silica‐coated aluminum oxide (2.8 bar, 10 mm, 15 s), air abrasion with 30 μm silica‐coated aluminum oxide + primer with MDP	Adhesive resin (VisioBond)	Composite resin (Filtek Z250)	Bond strength (shear)	Yes	Air abrasion with silica‐coated aluminum oxide particles in combination with the use of a MDP‐containg primer rendered the highest bond strength value among groups
AlRabiah et al. (2017)	In vitro	*n* = 10	Lithium disilicate (IPS e.max)	—	Scotchbond Universal Adhesive, All‐Bond Universal, Futurabond U; with or without silane	5% Hydrofluoric acid (20 s)	Composite resin (Tetric Ceram)	Bond strength (shear)	Yes	The use of silane improved the results of the different universal adhesive systems
Arami et al. (2014)	In vitro	*n* = 10	Zirconia (ICE Zirkon)	No surface treatment	Air abrasion with 50 μm aluminum oxide (10 mm, 2.8 bar, 10 s), Er,Cr:YSGG laser (2 W, 10 s, 2940 nm, 10 Hz), Nd: YAG laser (1.5 W, 2 min, 1064 nm, 10 Hz)	Clearfil porcelain bond activator + Clearfil SE Bond primer	Composite resin (Clearfil AP‐X)	Bond strength (shear) and surface roughness analysis	No	A direct correlation between surface roughness and superior bond strength was not observed. Air abrasion proved to be more efficient than laser
Atala and Yeğin (2022)	In vitro	*n* = 20	Feldspathic (Vitablocs Mark II)	—	All‐Bond Universal (hydrofluoric acid + silane + adhesive system), Prime&Bond (hydrofluoric acid + silane + adhesive system), Clearfil Quick Universal (phosphoric acid + adhesive system), Premio Bond (silane + adhesive system), Optibond XTR (hydrofluoric acid + silane + primer + bond), Tokuyama Universal Bond (adhesive system)	4air abrasion with 50 μm aluminum oxide	4composite resin (Filtek Z250)	Bond strength (microtensile)	Yes	All universal adhesive systems tested rendered appropriate bond strength values
Ataol and Ergun (2018)	In vitro	*n* = 14	Leucite (IPS e.max Ceram), lithium disilicate (IPS e.max CAD), zirconia (IPS e.max Zir‐CAD) and zirconia‐reinforced lithium disilicate ceramic (Vita Suprinity)	Clearfil ceramic primer and silane + clearfil universal bond	9% Hydrofluoric acid (90 s), air abrasion with 50 μm aluminum oxide (20 s, 10 mm), Er,Cr:YSGG laser (3 W, 2.94 μm wavelength, 50 Hz, 140 μs, 45°, 1 mm)	Clearfil ceramic primer or silane + clearfil universal bond	Composite resin (Clearfil Majesty ES‐2)	Bond strength (shear) and surface topography analysis (SEM)	Yes	Hydrofluoric acid rendered the highest results for the feldspatic and lithium disilicate ceramics. In addition, air abrasion led to the achievement of superior results for zirconia
Ataol and Ergun (2018)	In vitro	*n* = 14	Feldspatic (Vita VM11), lithium disilicate (IPS e‐max CAD), zirconia‐reinforced lithium disilicate ceramic (Vita Suprinity) and zirconia (IPS e.max Zir‐CAD)	Clearfil ceramic primer and silane + clearfil universal bond	9% Hydrofluoric acid (90 s), air abrasion with 50 μm aluminum oxide (20 s, 10 mm), Er,Cr:YSGG laser (3 W, 2.94 μm wavelength, 50 Hz, 140 μs, 45°, 1 mm)	Clearfil ceramic primer or silane + clearfil universal bond	Composite resin (Clearfil Majesty ES‐2‐A2)	Bond strength (shear) and surface topography analysis (SEM)	Yes	The application of hydrofluoric acid and air abrasion rendered satisfactory repair bond strength values for each tested ceramic
Attia (2010)	In vitro	*n* = 20	Zirconia (Vita) and feldspathic ceramic (Vita VM7)	Non‐fractured restorations	Bur, air abrasion with 50 μm aluminum oxide (2.8 bar, 5 s, 10 mm) and air abrasion with 30 μm silica‐coated aluminum oxide (2.8 bar, 20 s, 10 mm)	Bond	Composite resin (Filtek Z250)	Fracture resistance	Yes	Cyclic loading fatigue reduced the fracture loads of all groups. The experimental group including air abrasion with silica‐coated aluminum oxide and silane was associated with the best results
Baiomy et al. (2020)	In vitro	*n* = 20	Zirconia (Bio ZX2 Zirconium) and feldspatic ceramic (Ceramco PFZ)	—	Air abrasion with 50 μm aluminum oxide (2.8 bar, 10 s), air abrasion with silica‐coated aluminum oxide (2.8 bar, 15 s), laser (3 W, 2780‐nm wavelength, 140 μs, 50 Hz, 50 s, 1 mm), laser + air abrasion with silica‐coated aluminum oxide	Phosphoric acid + Z prime (zirconia)/silane (feldspatic) + bond	Composite resin (Clearfil AP‐X composite)	Bond stregth (shear)	Yes	Air abrasion with silica‐coated aluminum oxide was the most effective pre‐treatment for both ceramics
Barchetta et al. (2021)	In vitro	*n* = 10	Zirconia (In‐Ceram 2000 YZ)	Adhesive system and silanization (air abrasion: 30 μm silica‐coated Al_2_O_3_)	Prophylaxis, alcohol, and triple syringe	Adhesive system or silicatization	Composite resin (Opallis shade A2E)	Bond strength (shear), surface roughness analysis and surface topography analysis (SEM)	Yes	Zirconia should not be exposed in the oral cavity for a long period of time. Ideally, contaminated zirconia should be treated with prophylaxis (pumice stoneand water) or a triple syringe (waterand air jet), followed by silicatization and silane treatment, as to not affect bonding procedures
Barragan et al. (2014)	In vitro	*n* = 10	Zirconia (Lava Frame Zirconia)	No surface treatment and no primer	Air abrasion with 50 μm aluminum oxide (10 mm, 5 s, 40 psi); Monobond Plus, Z‐Prime Plus, and Experimental Zirkon‐Primer	—	Composite resin (Tetric Evoceram)	Bond strength (shear)	Yes	Air abrasion and the use of Z‐Prime Plus increased the shear bond strength of the resin composite to zirconia
Barutcigil and Kirmali (2020)	In vitro	*n* = 10	Lithium disilicate (e.max Press)	No surface treatment	9.6% Hydrofluoric acid (60 s), 37% phosphoric acid (60 s), Er Cr YSGG laser at 1, 2, and 3 W (2.78 μm wavelength, 10 Hz, 10 mm, 20 s)	Single‐bond universal adhesive	Composite resin (Grandio DC)	Bond strength (shear), EDS analysis, and surface topography analysis (SEM)	Yes	Hydrofluoric acid and the laser irradiation at 3 W rendered adequated bond strength values
Bayraktar et al. (2021)	In vitro	*n* = 2	Feldpatic ceramic (Vita Mark II)	—	Bur (15 s), 9% hydrofluoric acid (60 s), Nd:YAG laser (1064 nm, 1 mm, 3 W, 20 Hz, 200 μm, 20 s). Er:YAG laser (2940 nm, 1 mm, 3 W, 20 Hz, 300 μm, 20 s), ErCr:YSGG laser (2780 nm, 1 mm, 3 W, 20 Hz, 140 μm, 20 s)	Silane + universal bonding agent (Single Bond Universal)	Composite resin (Filtek Z250)	Bond strength (microtensile) and surface topography analysis (SEM)	Yes	Hydrofluoric acid is the most effective method for the felsdpatic ceramic
Benli et al. (2022)	In vitro	*n* = 20	Zirconia‐reinforced lithium silicate ceramic (Celtra Duo)	—	4.9% Hydrofluoric acid, Nd:YAG laser (150 mJ, 20 Hz, 3 W, 150 μs pulse duration, 1064 nm), Nd:YAG laser + hydrofluoric acid, Er:YAG laser (400 mJ, 10 Hz, 4 W, 100 μs pulse duration, 2940 nm), Er:YAG laser + hydrofluoric acid	Silane	Composite resin	Bond strength (shear), surface roughness analysis and surface topography analysis (SEM)	No	The association of hydrofluoric acid and Nd:YAG laser yelded the highest bond strength values and significantly increased surface roughness
Bergoli et al. (2016)	In vitro	*n* = 8	Feldspathis ceramic (Hera Ceram)	No surface treatment and 10% hydrofluoric acid (20 s)	Abrasion with rubber tips (40 s)	Silane + adhesive	Composite resin (Charisma)	Bond strength (microtensile), surface roughness and surface topography analysis (SEM)	Yes	The rubber abrasive tips presented results similar to the hydrofluoric acid control
Bessa et al. (2025)	In vitro	*n* = 15	Lithium disilicate (Smile‐lithium)	—	Cleaning with water, Ivoclean cleaning paste and air abrasion with 50 μm aluminum oxide (20 s, 10 mm, 2.5 bar, 45°); 5% hydrofluoric acid (20 s) + silane + adhesive and Monobond Etch&Prime	—	Composite resin (Filtek Z350)	Bond strength (shear) and surface topography analysis (SEM)	Yes	Air abrasion and Ivoclean provided effective cleaning for lithium disilicate ceramic prior to repair. Monobond Etch&Prime showed similar results to hydrofluoric acid followed by silane
Blum et al. (2012)	In vitro	*n* = 40	Leucite (Cerana insert)	No surface conditioning and no adhesive system applied	Ceramic Repair (diamond bur + total etch gel + monobond + heliobond), Cimara (bur + haftsilan + opaquer liquid), Clearfil Repair (diamond bur, etchant gel, mixed Clearfil SE Bond Primer and activator, Clearfil SE Bond), CoJet system (air abrasion with silica‐coated aluminum oxide + visio bond)	—	Composite resin (Nanosit for Clearfil Repair and CoJet system; Tetric EvoCeram for Ceramic repair; Arabesk for Cimara)	Bond strength (microtensile) and surface topography analysis (SEM)	No	The best bonding performance was obtained when the veneering ceramic was pre‐treated with hydrofluoric acid and the zirconia air abraded with silica coated aluminum oxide particles. Ideally, the veneer ceramic should be conditioned previously to the zirconia treatment
Çağlayan et al. (2025)	In vitro	*n* = 10	Lithium disilicate (CEREC Tessera) and zirconia (Lithoz and INNI‐Cer)	—	Clearfil Majesty Posterior or Filtek Z350	Zirconia: air abrasion with 50 μm aluminum oxide (10 s, 10 mm, 2.5 bar) and lithium disilicate: 9% hydrofluoric acid (20 s); adhesive system (Tokuyama Bond Force II)	Composite resin (Clearfil Majesty Posterior or Filtek Z350)	Color measurement	Yes	Lithium disilicate demonstrated the most consistent color stability
Carrabba et al. (2017)	In vitro	*n* = 10	Feldspathic ceramic (Vitablocs Mark II)	Porcelain Repair Kit group and CoJet + Premise Flow group	Air abrasion with 100 μm aluminum oxide (20 s, 4 atm), 9% hydrofluoric acid (120 s), air abrasion + hydrofluoric acid; Vertise Flow, Premise Flow, Optibond Solo Plus + Premise Flow	—	Flowable resin composite (Vertise Flow or Premise Flow)	Bond strength (shear) and surface topography analysis (SEM)	No	The results of this study show that the use of hydrofluoric acid is the most adequate treatment for feldspathic ceramic, while air abrasion did not significantly influenced bond strength values
Chaharom et al. (2018)	In vitro	*n* = 17	Lithium disilicate (IPS e.max Press)	No surface treatment	9.5% Hydrofluoric acid (60 s), Nd:YAG laser (30 mJ, 4.5 W, 15 Hz, 1.064 μm, 1 mm, 60 s), Nd:YAG laser (300 mJ, 6 W, 20 Hz, 1.064 μm, 1 mm, 60 s), Er,Cr:YSGG laser (300 mJ, 1.5 W, 2.78 μm, 1 mm, 60 s), Er,Cr:YSGG laser (300 mJ, 6 W, 2.78 μm, 1 mm, 60 s)	Silane + adhesive system (Silorane System Adhesive)	Composite resin	Bond strength (shear)	Yes	Hydrofluoric acid was associated with higher repair bond strength values in comparison the experimental groups using laser
Chen et al. (2010)	In vitro	NR	Feldspathic ceramic (GN‐I Ceramic Block)	Air abrasion	Carbon dioxide laser (30 mm), primer/silane and primer/silane + carbon dioxide laser (30 mm)	Air abrasion with 50 μm aluminum oxide (0.2 MPa, 15 s, 20 mm)	Composite resin (Lite‐Fil II A)	Bond strength (shear), Raman spectroscopy, and surface roughness analysis	Yes	The simultaneous use of silane and carbon dioxide laser led to superior shear bond strength between composite resin and ceramic
Çınar and Kırmalı (2019)	In vitro	*n* = 15	Zirconia (Nacera), feldspathic ceramic (Noritake CZR), and zirconia with feldspathic ceramic	No aging process	3000, 6000, and 12,000 thermal cycles	Clearfil Repair Kit: diamond bur, phosphoric acid, Clearfil SE Bond	Composite resin (Gradio SO)	Bond strength (shear)	Yes	Thermal cycles reduced the shear bond strength results. Zirconia rendered higher repair bond strength in comparison to the feldspathic veneer ceramic
Colares et al. (2013)	In vitro	*n* = 5	Lithium disilicate (IPS e.max)	—	9.5% Hydrofluoric acid (20 s) + silane (dried at 23°C), hydrofluoric acid + silane (dried with warm air at 45°C ± 5°C), air abrasion with 50 μm aluminum oxide (5 s, 30 psi, 10 mm) + silane (dried at 23°C), air abrasion with 50 μm aluminum oxide (5 s, 30 psi, 10 mm) + silane (dried with warm air at 45°C ± 5°C)	Silane (Rely‐X Ceramic Primer) and adhesive system (Adper Single Bond 2)	Composite resin (Filtek Z250)	Bond strength (microtensile)	No	Hydrofluoric acid resulted in adequate repair bond strength values. On the other hand, air abrasion should be avoided for lithium disilicate repair
Corazza et al. (2015)	In vitro	*n* = 6	Feldspathic ceramic (Vita Mark II)	Non‐aged groups	Blocks were thermocycled and sticks were obtained, sticks were obtained and then subjected to thermocycling, blocks were thermocycled, stored for 6 months at 37°C, and then sticks were obtained, and sticks were obtained and then subjected to thermocycling and stored for 6 months at 37°C	Hydrofluoric acid (10%, 60 s) + silane (Rely X Ceramic Primer) + adhesive (2.0 Adper Single Bond)	Composite resin (A2 TPH)	Bond strength (microtensile) and surface topography analysis (SEM)	Yes	The aging process significantly influenced the repair bond strength
Cristoforides et al. (2012)	In vitro	*n* = 10	Zirconia (in Ceram)	96% Isopropanol (with and without resin cement containing MDP)	Air abrasion with 30 μm silica‐coated aluminum oxide (2.8 bar, 10 mm, 15 s) + silane + resin cement with MDP, air abrasion with silica‐coated aluminum oxide + silane, 37% phosphoric acid + Clearfil SE Primer + Porcelain Bond Activator + resin cement with MDP, 37% OP acid + Clearfil SE Primer + Porcelain Bond Activator, air abrasion with 50 μm aluminum oxide (2.8 bar, 10 mm, 15 s) + silane + resin cement with MDP, air abrasion with aluminum oxide + silane, metal/zirconia primer + resin cement with MDP, metal/zirconia primer	—	Composite resin (Clearfil Majesty Esthetic)	Bond strength (shear)	Yes	The use of a resin cement containing MDP was not very effective. Air abrasion with silica‐coated aluminum oxide proved to be the most efficient strategy to zirconia repair with composite resin
Cunha et al. (2022)	In vitro	*n* = 4	Zirconia‐reinforced lithium disilicate ceramic (Vita Suprinity) and feldspathic ceramic (CEREM blocs)	—	5% Hydrofluoric acid—20, 40, and 60 s	Adhesive system (Scotchbond Universal)	Composite resin (Filtek 350 XT)	Bond strength (microtensile) and surface topography analysis (AFM)	No	The 40‐s period of etching with hydrofluoric acid was the most effective for the lithium disilicate. For the feldspathic ceramic, all time periods resulted in statistically similar results
de Melo et al. (2007)	In vitro	*n* = 5	Leucite (Omega 900)	—	6% Hydrofluoric acid (60 s) and air abrasion with 30 μm silica‐coated aluminum oxide (10 mm, 20 s, 2.8 bar)	Silane (Porcelain Primer) + adhesive system (Single Bond)	Composite resin (Filtek Z250)	Bond strength (microtensile) and surface topography analysis (SEM)	No	Both tested strategies provided similar bond strength values
Degirmenci et al. (2021)	In vitro	*n* = 15	Lithium disilicate (IPS e.max), leucite (IPS Empress) and feldspathic ceramic (Cerec Blocs)	Diamond bur	40% Phosphoric acid, air abrasion with 30 μm silica‐coated aluminum oxide (15 s, 2.5 bar, 10 mm), Er,Cr:YSGG laser (27 μm wavelength, 4 W, 14 Hz)	Silane (G‐Multi Primer) + universal adhesive system (G‐Premio Bond)	Composite resin (G‐aenial Antrerior)	Bond strength (shear) and surface topography analysis (SEM)	Yes	Laser irradiation enhanced the repair bond strength in leucite‐reinforced and feldspathic ceramics. On the other hand, for lithium disilicate the application of acid‐etching is preferable
Della‐Bona (2005)	Review	Roughening the ceramic surface by hydrofluoric acid etching and silane coating yields the highest bond strength values for acid‐sensitive ceramics. Silica coating acid‐resistant ceramics is important to improve bonding to resin. The microtensile test may be preferable to conventional shear or flexural tests as an indicator of composite‐ceramic bond quality								
Dos Santos et al. (2019)	In vitro	*n* = 10	Zirconia (IPS e.max ZirCAD)	Z‐Prime Plus and air abrasion with 50 μm aluminum oxide (15 s) + Z‐Prime Plus	Single Bond Universal, All Bond Universal, Z‐Prime Plus + All Bond Universal, air abrasion with 50 μm aluminum oxide (15 s) + Single Bond Universal, air abrasion with 50 μm aluminum oxide (15 s) + All Bond Universal and air abrasion with 50 μm aluminum oxide (15 s) + Z‐Prime Plus + All Bond Universal	—	Composite resin (Filtek Z350)	Bond strength (shear) and surface topography analysis (SEM)	No	The present study demonstrated that air abrasion is crucial for the process of zirconia repair
Duzyol et al. (2015)	In vitro	*n* = 5	Feldspathic ceramic (Cerec Blocs) and lithium disilicate (IPS e.max)	Bur	5% Hydrofluoric acid (5 min), air abrasion with 50 μm aluminum oxide (10 s, 5 mm) and air abrasion with 30 μm silica‐coated aluminum oxide (10 s, 5 mm)	Bur + silane (Rely X Ceramic Primer) + adhesive system (Single Bond Universal)	Composite resin (Filtek Z 550)	Bond strength (microtensile)	Yes	The use of hydrofluoric acid was the most efficient pre‐treatment for lithium disilicate. Any of the pre‐treatements rendered superior results for feldspathic and hybrid ceramics, in comparison to the control groups where bur was used
Elraggal and Silikas (2021)	In vitro	*n* = 30	Zirconia (IPS e. max Zir‐CAD)	No surface treatment	Air abrasion with 30 μm silica‐coated aluminum oxide (3 bar, 15 s, 10 mm), air abrasion with fluorapatite glass–ceramic (FGC) (3 bar, 15 s, 10 mm) and laser (4 W, 20 Hz, 180 μm, 1 min, 1 mm)	Monobond Plus + adhesive system (Adhese Universal) for the composite resin and resin cement (Multilink Automix) for the ceramics	Composite resin (Tetric Evocerma Bulk Fill), feldspathic ceramic (VITAVM 13) or lithium disilicate (IPS e.max)	Bond strength (shear), crystallinity, surface roughness analysis, EDS analysis and surface topography analysis (SEM)	Yes	The group with laser rendered superior surface roughness in comparison to the air abrasion groups. Air abrasion with FGC provided a superior amount of silica than air abrasion with silica‐coated aluminum oxide
Elraggal et al. (2022)	In vitro	*n* = 40	Zirconia (IPS e.max ZirCAD)	No surface treatment	Air abrasion with silica‐coated aluminum oxide (3 bar, 15 s, 10 mm) and fluorapatite glass–ceramic (FGC) (3 bar, 15 s, 10 mm); Monobond Plus, Calibra Silane 2, Scotchbond Universal, Futurabond + resin cement (Multikink Automix)	—	Composite resin (Tetric Evoceram Bulk Fill)	Bond strength (shear), surface roughness analysis, EDS analysis and surface topography analysis (SEM)	Yes	The MDP‐containing adhesive enhanced the bond strength values. Air abrasion with FGC rendered superior results than silica‐coated aluminum oxide
Erdemir et al. (2014)	In vitro	*n* = 26	Lithium disilicate (IPS e.max)	No surface treatment	Air abrasion with 30 μm silica‐coated aluminum oxide (2.5 bar, 10 mm, 10 s), 9.6% hydrofluoric acid (20 s), laser (2.940 nm, 300 mJ, 20 Hz, 6 W, 1 mm, 20 s) and diamond bur (10 s)	Silane	Flowable composite resin (Vertise Flow)	Bond strength (shear) and surface topography analysis (FE‐SEM)	No	Laser and the diamond bur rendered inferior repair bond strength values in comparison to hydrofluoric acid and air abrasion
Falah and Ameer (2020)	In vitro	*n* = 8	Feldspathic (Cerec block) and lithium disilicate (IPS e.max)	—	Ceramic repair kit, Scothcond universal adhesive and Palfique universal bond	—	Composite resin (Tetric EvoCeram)	Bond strength (shear)	No	Palfique universal bond was associated with the best results for the lithium disilicate. Scothbond universal performed better for the lithium disilicate
Fathpour et al. (2023)	In vitro	*n* = 20	Zirconia (IPS e.max zirCAD)	—	Air abrasion + Z‐Prime Plus, bur + Z‐Prime Plus, air abrasion + Z‐Prime Plus with G premio bond, bur + Z‐Prime Plus with G premio bond, air abrasion + Z‐Prime Plus with All Bond Universal, bur + Z‐Prime Plus with All Bond Universal, air abrasion + Z‐Prime Plus with Clearfil SE bond, bur + Z‐Prime Plus with Clearfil SE bond, air abrasion + G‐Premio Bond, bur + G‐Premio Bond, air abrasion + All Bond Universal, bur + All Bond Universal, air abrasion + Clearfil SE Bond, bur + Clearfil SE Bond	—	Composite resin (Charisma Diamond)	Bond strength (shear)	Yes	Air abrasion proved to be more efficient than bur roughneing. The experimental groups of Clearfil SE Bond and Z‐Prime Plus + Clearfil SE Bond achieved the best results
Flury et al. (2019)	In vitro	*n* = 15	Feldspathic (Vitablocs Mark II)	—	Monobond Plus (silane) + Optibond FL adhesive and Scotchbond Universal	Air abrasion with aluminum oxide and silica‐coated aluminum oxide	Composite resin (Filtek Z250)	Bond strength (shear)	Yes	For restoration repair, optimal bond strength values are obtained when a silane and a bonding agent are both used after air abrasion with aluminum oxide
Galvão Ribeiro et al. (2018)	In vitro	*n* = 28	Zirconia (Lava Zirconia)	—	Air abrasion with 50 μm aluminum oxide (15 s, 0.2 MPa, 10 mm) and air abrasion with 30 μm silica‐coated aluminum oxide; Clearfil SE Bond Primer + Clearfil Porcelain Bond Activator and Rely X primer + Rely X resin cement	—	Composite resin (Filtek Z350)	Bond strength (shear)	Yes	The association of air abrasion with silica‐coated aluminum oxide and Rely X rendered the highest repair bond strength values
Gençer et al. (2025)	In vitro	*n* = 15	Leucite (IPS Empress)	—	Air abrasion with 50 μm aluminum oxide, 30 μm silica‐coated aluminum oxide or 27 μm bioactive glass (10 s, 15 mm, 2.5 bar); two‐ (Tokuyama Universal) or one‐bottle (G‐Premio) universal adhesive	—	Composite resin (Omnichroma or Essentia Universal)	Bond strength (shear), surface roughness and surface topography analysis (SEM)	No	Bioactive‐glass and silica‐coated alumina caused similar surface roughness and led to similar bond strength values
Ghavam et al. (2017)	In vitro	*n* = 10	Zirconia (Kuraray Noritake) and feldspathic ceramic (Ceramico)	No surface treatment	Zirconia: Er,Cr:YSGG laser, air abrasion + Z‐Primer Plus, air abrasion + Z‐Primer Plus; feldspathic: Er,Cr:YSGG laser, air abrasion + 9.5% hydrofluoric acid + silane, Er,Cr:YSGG laser + hydrofluoric acid + silane	—	Composite resin (Vertise Flow)	Bond strength (shear)	Yes	The use of laser produces results similar to those of the hydrofluoric acid and air abrasion experimental groups
Goia et al. (2006)	In vitro	*n* = 10	Alumina reinforced feldspathic ceramic (Vitadur‐α)	—	9.6% hydrofluoric acid + silane, air abrasion with 110 μm aluminum oxide + silane, air abrasion with 30 μm silica‐coated aluminum oxide + silane	Adhesive system (Single Bond)	Composite resin (W3D‐Master)	Bond strength (microtensile) and surface topography analysis (SEM)	No	Hydrofluoric acid was associated with higher repair bond strength values than air abrasion
Gouda et al. (2023)	In vitro	*n* = 6	Zirconia (Wieland Dental)	No surface treatment	Air abrasion with 110 μm aluminum oxide and diamond bur; All Bond Universal, Z prime plus and TheraCem	—	Composite resin (Polofil NHT)	Bond strength (shear) and surface topography analysis (SEM)	No	Bur led to superior results in comparison to air abrasion. The chemical pretreatments containing 10‐MDP provided higher bond strength values
Guler et al. (2005)	In vitro	*n* = 12	Feldspathic ceramic (Vita VMK95)	—	Air abrasion with 50 μm aluminum oxide, air abrasion with 110 μm aluminum oxide, 9.6% hydrofluoric acid, silane, air abrasion with 50 μm aluminum oxide + silane, air abrasion with 110 μm aluminum oxide + silane, hydrofluoric acid + silane, air abrasion with 50 μm aluminum oxide + hydrofluoric acid + silane, air abrasion with 110 μm aluminum oxide + hydrofluoric acid + silane	Adhesive system (Prime&Bond NT)	Composite resin (Arabesk Top)	Bond strength (shear)	No	The experimental group including air abrasion with 50 μm particles, hydrofluoric acid and silane achieved the highest bond strength values
Güler et al. (2006)	In vitro	*n* = 14	Feldspathic ceramic (Vita VMK95)	No surface treatment	Hydrofluoric acid (9.6%) for 30, 2 × 30, 60, 2 × 60, 120, and 180 s; one step adhesive and self‐etching adhesive system	—	Composite resin (Filtek Z250)	Bond strength (shear)	No	There was no difference between the 60 and the 2 × 30 s group, as well as between 120 and 2 × 60 s. Single Bond adhesive performed better than the self‐etching adhesive system
Güler et al. (2009)	In vitro	*n* = 10	Feldspathic ceramic (Vita MVK95)	—	Adper Prompt L‐Pop, Quadrant Uni 1 Bond, Te‐Econom, PQ 1, One Step Plus and Prime&Bond NT	Air abrasion with 50 μm Al2O3 + hydrofluoric acid (9.6%) + silane (Silane Bond Enhancer)	Composite resin (Filtek Z250)	Bond strength (shear)	Yes	Adhesive systems with a high filler ratio rendered better results
Habib et al. (2021)	In vitro	*n* = 15	Zirconia (ZirCAD) with veneer feldspathic ceramic (IPS Ceram e‐max)	—	9.5% Hydrofluoric acid + porcelain primer + Z‐Prime Plus + bonding resin, Monobond + Heliobond, Signum ceramic bond I and II, Scoth Bond, and Single Bond	—	Composite resin (Tetric Evoceram)	Bond strength (shear) and surface topography analysis (SEM)	Yes	The complete ceramic/zirconia repair systems showed better bond strength between the repaired composite and zirconia core
Hakimaneh et al. (2020)	In vitro	*n* = 10	Feldspathic	—	11% Hydrofluoric acid + silane, silane + CO_2_ laser, CO_2_ laser + silane, silane + Er:YAG laser, Er:YAG laser + silane, bur + hydrofluoric acid + silane	Adhesive resin	Composite resin (Tetric N‐Ceram)	Bond strength (shear)	Yes	Laser is only effective when applied prior to silane, with results similar to those obtained by the combination of hydrofluoric acid and silane
Han et al. (2013)	In vitro	*n* = 10	Zirconia	—	Air abrasion with 30 μm silica‐coated aluminum oxide + silane based primer (Rely X) + Adper Single Bond, Ceramic Repair System (zirconia primer + Heliobond), Signum Zirconia Bond	Diamond bur	Composite resin (Filtek Z350)	Bond strength (shear), surface roughness analysis, wettability and surface topography analysis (FE‐SEM)	No	Signum Zirconia Bond and air abrasion with silica‐coated aluminum oxide rendered the highest bond strength values
Hassan et al. (2023)	In vitro	*n* = 11	Feldspathic ceramic (DeguDent)	Hydrofluoric acid (9.5%)	CO_2_ laser, CO_2_ laser + hydrofluoric acid	Porcelain primer and porcelain bonding resin	Composite resin (Trtriv N‐Ceram)	Bond strength (shear), FTIR test, EDS analysis, surface roughness analysis and surface topography analysis (SEM)	No	The subsequent use of laser and hydrofluoric acid led to the highest repair bond strength values
Höller et al. (2022)	In vitro	*n* = 30	Lithium disilicate (IPS e.max)	No surface treatment	Air abrasion with aluminum oxide + silane and primer; laboratory, rubber dam, and oral conditions	Bonding resin (Heliobond) and composire resin as the luting agent	Lithium disilicate (IPS e.max)	Bond strength (microtensile)	Yes	The use of the Monobond Etch&Prime performed better than the experimental group including air abrasion and silane. Higher bond strength values were achieved when the environment had minimum humidity
Huang et al. (2013)	In vitro	*n* = 15	Lithium disilicate (IPS Empress 2)	—	Diamond bur and air abrasion with silica‐coated aluminum oxide; 37% phosphoric acid and 9.6% hydrofluoric acid; silane or not	Adhesive resin (XP Bond)	Composite resin (Ceram X Mono)	Bond strength (microtensile) and surface topography analysis (SEM)	Yes	Hydrofluoric acid was the most effective pretreatment, independently of silanisation and surface mechanical treatments
Hwang et al. (2018)	In vitro	*n* = 5	Leucite (IPS e. max Ceram)	—	Vitablocs Mark II (feldspathic), IPS Empress (leucite), IPS e‐max (lithium disilicate), Vita Enamic (hybrid) and composite resin (Filtek Z250)	5% Hydrofluoric acid + silane + bonding resin + resin cement	—	Bond strength (microtensile) and surface roughness analysis	Yes	Repair procedures resorting to ceramic in alternative to composite resin provided superior bond strength results
Janson et al. (2025)	In vitro	*n* = 72	Zirconia (Katana Zirconia HT)	No surface treatment	Air abrasion with 50 μm aluminum oxide (10 s, 2 bar), air abrasion with 110 μm aluminum oxide (10 s, 2 bar); Optibond Universal, Prime&Bond Active, iBond Universal, Clearfil Universal Bond Quick, Monobond Plus and Scotchbond Universal Plus	—	Composite resin (Clearfil Majesty ES‐2 Universal)	Bond strength (shear) and surface topography analysis (SEM)	Yes	Air abrasion of zirconia enhances the bond strength of repair composites. Universal adhesives containing MDP exhibit superior bond strength values
Kameyama et al. (2018)	In vitro	*n* = 8	Leucite (IPS Empress CAD)	40% Phosphoric acid (5 s)	Clearfil SE Bond primer + Clearfil Porcelain Bond Activator, atmospheric‐pressure plasma (APP) irradiation, UV irradiation, APP + Clearfil SE Bond primer + Clearfil Porcelain Bond, UV + Clearfil SE Bond primer + Clearfil Porcelain Bond	40% Phosphoric acid (5 s) + Clearfil SE Bond	Composite resin (Herculite XRV)	Bond strength (microtensile) and wettability	No	The application of phosphoric acid prior to silane led to enhanced repair bond strength values and water contact angle. Both irradiation treatments did not affect the results
Kanzow et al. (2019)	Systematic review	The main treatment steps were consistently reported across repair protocols. The included protocols were mainly based on studies with low level of evidence								
Karabulut Gencer et al. (2023)	In vitro	*n* = 10	Leucite (IPS Empress)	—	Air abrasion with 30 μm silica‐coated aluminum oxide, 27 μm bioactive glass and 50 μm aluminum oxide	Tokuyama Universal Bond or Multiprimer + G‐premio Bond	Composite resin (Omnichroma or Essentia)	Color measurement	Yes	Surface preparation did not significantly affect color stability
Karcı et al. (2018)	In vitro	*n* = 9	Leucite (IPS Empress) and lithium disilicate (IPS e.max)	—	Ceramic Repair system (Monobond + Heliobond) and Nova Compo SF	5% Hydrofluoric acid	Composite resin (Tetric Evoceram) for Ceramic Repair system and flowable composite for Nova Compo SF	Bond strength (shear)	Yes	The two tested repair systems present statistically similar repair bond strength values
Kazak et al. (2019)	In vitro	*n* = 8	Leucite (IPS Empress)	Distilled water	Pomegranate‐flavored mineral water (pH 3.39), salad dressing with balsamic vinegar + olive oil + lemon + pomegranate molasses + basil + salt + and garlic (pH 2.30), for 24 h	5% Hydrofluoric acid (60 S) + silane (Monobond) + bonding agent (Heliobond)	Composite resin (Filtek Z550, Aelite Esthetic Enamel, Tetric N‐Ceram Bulk‐ Fill, Clearfil Majesty Esthetic)	Bond strength (shear) and color measurement	No	Both tested liquids, led to enhanced color changes and surface degradation than the control group with distilled water. Among the materials, Aelite Esthetic Enamel displayed the least color change due to its filler composition and distribution
Kilinc et al. (2020)	In vitro	*n* = 11	Lithium disilicate (IPS e.max) and feldspathic ceramic (Vita Mark II)	No surface treatment	Air abrasion with 50 μm aluminum oxide (10 mm, 3 bar, 20 s), 5% hydrofluoric acid, Er,Cr:YSGG laser (2780 nm, 2 W, 20 mHz, 140 μs, 5 mm, 40 s)	Bonding agent (Single Bond Universal Adhesive)	Composite resin (Filtek Z550)	Bond strength (shear) and surface topography analysis (SEM)	No	Hydrofluoric acid is the most effective pre‐treatment for glass ceramics. The aging process affected the repair bond strength values obtained
Kim et al. (2005)	In vitro	*n* = 10	Feldspathic (DuceramPlus), lithium disilicate (IPSEmpress2), alumina (In‐Ceram Alumina) and zirconia (Zi‐Ceram)	Feldspathic ceramic	Air abrasion with 50 μm aluminum oxide (40 psi, 5 s), air abrasion with 50 μm aluminum oxide (40 psi, 5 s) + 4% hydrofluoric acid (5 min) and air abrasion with 30 μm silica‐coated aluminum oxide (40 psi, 5 s)	Silane and bonding agent (One‐Step)	Composite resin (Z100 Restorative)	Bond strength (microtensile) and surface topography analysis (SEM)	No	For alumina and zirconia, air abrasion with silica‐coated aluminum oxide was the most efficient pre‐treatment. On the other hand, the use of air abrasion with aluminum oxide, followed by the hydrofluoric acid rendered the highest bond strength values for glass ceramics
Kim et al. (2007)	In vitro	*n* = 5	Leucite (IPS d.SIGN)—A2, A3 and A3.5	—	Different shades of composite resin	—	Composite resin (Arabesk Top, Filtek Supreme and Tetric Ceram)	Color measurement	No	Metameric effects varied depending on porcelain shade, resin composite brand, and illuminant, with interactions between these factors
Kimmich and Stappert (2013)	Restorations should be treated on the basis of the material that is exposed on the fractured surface. When presented with metal oxide ceramics the CoJet system should be used, followed by the application of a silane and a phosphate monomer. On the other hand, silicate ceramics should be etched with hydrofluoric acid or sandblasted. The application of a silane afterward is crucial to achieve a chemical bond between the resin and the ceramic material									
Kiomarsi et al. (2022)	In vitro	*n* = 10	Leucite (IPS Empress)	—	Laser irradation (2 W, 200 mJ, 10 Hz, 1 mm, 10 s) and 90% buffered hydrofluoric acid (60 s); silane and no silane; universal adhesive (Scotchbond Universal Adhesive) and conventional adhesive system (AdperScotchbond)	—	Composite resin (Filtek Z250)	Bond strength (shear)	No	Hydrofluoric acid rendered superior results in comparison to laser. The combination of hydrofluoric acid, silane and a conventional adhesive system led to satisfactory results
Kirmali et al. (2015)	In vitro	*n* = 10	Zirconia (Y‐TZP Noritake Alliance)	Bur	Air abrasion with 30 μm silica‐coated aluminum oxide (20 s, 10 mm), Nd:YAG laser (1.064 lm, 20 Hz, 200 mJ, 1 W, 300 ms, 20 s, 1 mm), Er,Cr:YSGG laser (2.78 μm, 20 Hz, 0.25–6 W, 20 s, 10 mm), air abrasion with 30 μm silica‐coated aluminum oxide + Nd:YAG laser, air abrasion with 30 μm silica‐coated aluminum oxide + Er,Cr:YSGG laser	Cimara repair system (silane + opaquer liquid)	Composite resin (Grandio)	Bond strength (shear) and surface topography analysis (SEM)	no	The combination of laser and air abrasion enhanced the bond strength for zirconia repair
Kirmali et al. (2015)	In vitro	*n* = 10	Zirconia (Y‐TZP Noritake Alliance)	No surface treatment	Air abrasion with 30 μm silica‐coated aluminum oxide, Cimara System (cimara bur + silane + opaquer liquid), Z‐Prime Plus System (primer), Clearfil System (etchant + Clearfil SE Bond Primer and activator + Clearfil SE Bond) and Z‐Bond System (primer)	Diamond bur	Composite resin (Grandio)	Bond strength (shear)	No	Cimara System rendered the highest bond strength values
Kocaağaoğlu et al. (2017)	In vitro	*n* = 10	Zirconia (Zirkozahn SRL), alumina (In‐Ceram Alumina) and feldspathic ceramic (Vitablocks Mark II)	—	Bisco Intraoral Repair Kıt, Cimara & Cimara Zircon Repair System and Clearfil Repair System	—	Composite resin (GrandioSO)	Bond strength (shear) and wettability	Yes	The Cimara repair systems was associated with the highest bond strength values
Kukiattrakoon and Thammasitboon (2007)	In vitro	*n* = 10	Leucite (IPS Empress)	no surface treatment	1.23% APF gel (New Age Fluoride Gel) for 1, 2, 3, 4, 5, 6, 7, 8, 9 and 10 min and 9.6% hydrofluoric acid for 4 min	Silane (Monobond‐S), primer (Scotchbond Multi‐Purpose Plus primer) and bonding agent (Scotchbond Multi‐Purpose Plus adhesive)	Composite resin (Filtek Z250)	Bond strength (shear) and surface topography analysis (SEM)	No	The application of hydrofluoric acid rendered similar results than 1.23% APF gel applied for 7, 8, 9 and 10 min
Külünk and Saraç (2009)	In vitro	*n* = 8	Lithium disilicate (IPS Empress 2) and leucite (IPS Empress Esthetic)	—	Ceramic repair kit (37% OP acid for 15 s, silane and adhesive resin), Clearfil repair (40% phosphoric acid, Clearfil Porcelain Bond Activator, primer and bond of Clearfil SE Bond) and air abrasion with 30 μm silica‐coated aluminum oxide (10 mm, 15 s, 3 psi, 90°) + silane + adhesive resin	—	Composite resin (Tetric Ceram)	Bond strength (shear), surface roughness analysis, wettability and surface topography analysis (SEM)	Yes	The use of phosphoric acid proved to be insufficient to achieve adequate bond strength values and significant alterations in surface morphology
Kumchai et al. (2020)	In vitro	*n* = 10	Feldspathic ceramic (Vita Trilux Forte)	—	Bonding agent (Heliobond) + Tetric EvoCeram, bonding agent (G‐aenial bond) + G‐aenial Evo Flo, silane + resin cement + feldspathic ceramic, silane + resin cement + lithium disilicate	5% Hydrofluoric acid (60 s)	Composite resin or ceramic (lithium disilicate and feldspathic ceramic)	Fracture resistance and surface topography analysis (SEM)	Yes	The experimental groups using ceramic as the repair material presented superior fracture resistance, with lithium disilicate to perform better than feldspathic ceramic
Lee (2007)	In vitro	*n* = 5	Leucite (IPS d.SIGN)—A2, A3 and A3.5	—	Different shades of composite resin	—	Composite resin (Arabesk Top, Filtek Supreme and Tetric Ceram)	Color measurement	Yes	The aging process influenced the translucency of the restoration, leading to a mismatch between the ceramic and the composite resin used for repair
Lee et al. (2014)	In vitro	*n* = 12	Feldspathic ceramic (Creation AV), zirconia (Lava Zirconia) and alumina (NobelProcera)	100% Veneering ceramic surface	100% Alumina surface, 100% zirconia surface, 50% alumina surface, 50% zirconia surface	Air abrasion with 30 μm aluminum oxide (2.5 bar), 35% phosphoric acid, silane primer (Rely X), adhesive system (Adper Single Bond PLus)	Composite resin (Filtek Supreme Plus)	Bond strength (shear)	No	Bond strength was superior for the veneer ceramic surface in comparison to alumina and zirconia, which presented similar results
Li et al. (2014)	In vitro	NR	Feldspathic ceramic (VITA VMK 95)	—	Two experimental silane coupling agents; opaque resin with light irradiation, opaque resin without light irradiation and no opaque resin	Clearfil Repair Multi‐Purpose repair kit (40% OP acid + primer with 10‐MDP and silane)	Composite resin (Clearfil Majesty Esthethic)	Bond strength (shear)	Yes	The highest bond strength results in the experimental groups with 30 mol% BTS with 0.05 mol/L hydrochloric acid in an MPTS‐BTS silane mixture, with a prolonged silanation period of 60 min
Li et al. (2020)	In vitro	*n* = 32	Zirconia (ZS‐B70)	—	Air abrasion with 50 μm aluminum oxide (0.25 MPa, 30 s, 20 mm) and no mechanical pretreatment; monobond and novel silane; Heliobond and Cleafil SE Bond	—	Composite resin (Filtek Z350)	Bond strength (shear) and surface topography analysis (SEM)	Yes	The association of air abrasion, novel silane and bonding agent containing MDP resulted in the highest repair bond strength values
Libecki et al. (2017)	In vitro	*n* = 16	Zirconia (Cercon)	Silicon carbide paper (negative control), air abrasion with 50 μm aluminum oxide (positive control)	Silicon carbide grinding bur	Primer (Cimara Zircon Primer), adheisve (Cimara Zircon Adhesive), dual‐curing adhesive resin (Bifix)	Self‐curing composite resin (Grandio Core Dual Cure)	Bond strength (microtensile), surface roughness analysis and surface topography analysis (SEM)	Yes	The use of air abrasion or silicon carbide bur led to adequate repair bond strength values
Lundvall et al. (2012)	In vitro	*n* = 10	Feldspathic ceramic (Hera‐ceram)	—	9% Hydrofluoric acid (60 s) + two‐part silane (Bis‐Silane) + adhesive resin (Porcelain Bonding Resin) + composite, 9% hydrofluoric acid (60 s) + adhesive resin + composite, 9% hydrofluoric acid (60 s) + one‐part silane (Silane) + adhesive resin + composite, 35% phosphoric acid + Bis‐silane + adhesive resin + composite, 15% HCl + Bis‐silane + adhesive resin + composite, 9% hydrofluoric acid (60 s) + Bis‐Silane + adhesive resin (Porcelain Bonding Resin) + composite cement (Choice) + ceramic, 15% potassium fluoride/hydrogen fluoride (KF·hydrofluoric acid) and carboxymethylcellulose + Bis‐silane, adhesive resin + composite	—	Composite resin (Charisma) or feldspathic ceramic	Bond strength (shear)	Yes	15% KF·hydrofluoric acid and 9% hydrofluoric acid led to similar bond strength values. The use of ceramic as the repair material rendered superior results in comparison to the use of composite resin
Maawadh et al. (2020)	In vitro	*n* = 10	Lithium disilicate (IPS e.max)	—	Er,Cr:YSGG laser (2790 nm, 30 Hz, 3.75 W, 140 μs, 120 s), photodynamic therapy using methylene blue photosensitizer (100 mg/L), 9.6% hydrofluoric acid (60 s) + silane, air abrasion with 30 μm silica‐coated aluminum oxide (10 s, 10 mm, 2 bar) + silane	Porcelain Repair Kit + adhesive system (Peak universal bond)	Composite resin (Clearfil Majesty Esthetic)	Bond strength (shear) and surface topography analysis (SEM)	Yes	The use of air abrasion or hydrofluoric acid rendered superior bond strength values in comparison to the use of photodynamic therapy
Mahgoli et al. (2019)	In vitro	*n* = 12	Zirconia (Cercon)	Porcelain Bonding resin	Z‐Prime Plus, MonoBond (silane), Z‐Prime Plus + Porcelain Bonding resin, MonoBond + Porcelain Bonding resin	—	Composite resin (Z100)	Bond strength (shear) and surface topography analysis (SEM)	Yes	Z‐Prime Plus was more effective to increase the repair bond strength values than the silane. The application of Porcelain Bonding resin potentiated the results
Matinlinna and Vallittu (2007)	The repair of a fractured ceramic with silica‐coated aluminum oxide, followed by a silane application is, according to the literature, a relevant adhesion promotion method in prosthetic dentistry									
Meshramkar and Sajjan (2010)	In vitro	*n* = 7	Feldspathic ceramic (Vita MK95)	—	Air abrasion with 50 μm aluminum oxide (10 s), 35% phosphoric acid, 8% hydrofluoric acid (4 min), air abrasion with 50 μm aluminum oxide + ceramic primer + Scotchbond adhesive, air abrasion with 50 μm aluminum oxide + Clearfil Liner Bond, 35% phosphoric acid + Scotchbond adhesive, 35% phosphoric acid + Clearfil Liner Bond, 8% hydrofluoric acid (4 min) + Scotchbond adhesive, 8% hydrofluoric acid (4 min) + Clearfil Liner Bond	—	Composite resin	Bond strength (shear)	No	Hydrofluoric acid led to superior results in comparison to air abrasion and phosphoric acid. Regarding the adhesive system, the Clearfill Liner Bond performed better than the Scothbond adhesive
Mohamed et al. (2014)	In vitro	*n* = 22	Feldspathic ceramic (GC Initial IQ)	No surface treatment	40% Phosphoric acid (5 s) + silane primer, < 5% hydrofluoric acid (60 s) + 40% phosphoric acid (5 s) + silane primer, air abrasion with 30 μm silica‐coated aluminum oxide (45 psi, 10 mm, 15 s, 90°) + 40% phosphoric acid (5 s) + silane primer; Clearfil Esthetic Cement and Panavia F 2.0 Cement	—	Feldspathic ceramic (GC Initial IQ): treated with < 5% hydrofluoric acid (60 s) + 40% phosphoric acid (5 s) + silane	Bond strength (shear)	Yes	The combination of hydrofluoric acid and phosphoric acid rendered the highest bond strength results. Clearfil Esthetic Cement was associated with lower values in comparison to Panavia F 2.0 Cement
Mohammadi et al. (2015)	In vitro	*n* = 26	Feldspathic (Ceramco)	—	Adper Single Bond, Clearfil SE Bond, Silorane Adhesive System	Air abrasion with 50 μm aluminum oxide (50 bar, 10 mm, 10 s) + 37% OP acid (2 min) + silane (Pulpdent)	Composite resin (Filtek Z250 or Filtek Silorane)	Bond strength (shear)	Yes	Although the type of composite resin did not affect the bond strength values, the use of Clearfil SE Bond was associated with the worst results, while Adper Single Bond 2 and Silorane Adhesive were similar between them
Moravej‐Salehi et al. (2016)	In vitro	*n* = 17	Feldspathic ceramic (Ceramco)	9.5% Hydrofluoric acid (2 min)	Air abrasion with 50 μm aluminum oxide (10 s, 5 mm, 90°) at 2, 3 and 4 bar	Silane (Bis‐Silane) + resin bond (D/E Resin)	Composite resin (AELITET All Purpose Body)	Bond strength (shear) and surface topography analysis (SEM)	Yes	No statistically significant difference was observed between the control and the three experimental groups
Naderi et al. (2025)	In vitro	*n* = 12	Feldspathis ceramic (VItaBlocks Mark II) and zirconia	No surface treatment	9% Hydrofluoric acid (60 s), air abrasion with 50 μm aluminum oxide (10 s, 10 mm, 2.5 bar, 90°), silicon carbide grinding (180 μm, 20 s)	Silane + adhesive (Clearfill Universal Bond)	Composite resin (Gradia‐Direct)	Bond strength (microtensile)	No	For the feldspathic ceramic, hydrofluoric acid rendered the highest bond strength values. For zirconia, the different surface treatments did not significantly improve repair bond strength compared to no treatment
Neis et al. (2015)	In vitro	*n* = 3	Feldspathic ceramic (Vita VM7), leucite (IPS Empress) and lithium disilicate (IPS e.max)	No surface treatment	Diamond bur, 10% hydrofluoric acid (90 s for feldspathic ceramic, 60 s for leucite, 20 s for lithium disilicate), air abrasion with 45 μm silica‐coated aluminum oxide (20 s, 10 mm, 90°, < 2.8 bar)	37% Phosphoric acid (30 s) + silane + adhesive system (Adapter Singlebond 2)	Composite resin (Filtek Z350)	Bond strength (microtensile)	Yes	Diamond burs are suitable for leucite and feldspathic ceramic. Hydrofluoric acid etching is recommended for lithium disilicate and air abrasion is effective for leucite repairs
Nogueira et al. (2023)	The combination of hydrofluoric acid, air abrasion with aluminum oxide or silica‐coated aluminum oxide, plus silane, and an adhesive, led to an improvement in the bond strength of composite resin‐repaired glass–ceramics. For feldspathic materials, air abrasion is an alternative method to hydrofluoric acid etching to improve micromechanical retention. Silane is an essential step, but adhesive is optional when silane is applied. For leucite, quantitative results suggest the use of silica‐coated aluminum oxide is comparable with the use of hydrofluoric acid. For lithium disilicate, alternatives to hydrofluoric acid were not found, but silane is an essential step									
Oh and Shen (2005)	In vitro	*n* = 1	Lithium disilicate (Eris) and leucite (IPS Empress)	No surface treatment	Air abrasion with 50 μm aluminum oxide (0.24 Mpa), 5% hydrofluoric acid (2 min), air abrasion + acid etching; steam and flame cleaning	Adhesive resin (Heliobond)	Composite resin (Tetric Ceram)	Bond strength (microtensile)	No	The process of flame cleaning increased the bond strength values
Ozcan et al. (2009)	In vitro	*n* = 10	Alumina‐reinforced feldspathic ceramics (Vitadur‐α)	—	Biscor Repair Kit: 9.5% hydrofluoric acid (60 s) + silane (Porcelain Primer) + adhesive (One‐Step), CoJet Repair Kit: air abrasion with 30 μm silica‐coated aluminum oxide + silane (ESPE‐Sil) + adhesive resin (Visio‐Bond), Clearfil Repair Kit: diamond bur + 40% phosphoric acid + Clearfil SE Bond Primer + Clearfil Porcelain Bond Activator	—	Composite resin (W3D‐Master)	Bond strength (microtensile)	Yes	The aging process did not interfere with the CoJet system, but instead influenced the bond strength values on Bisco Repair Kit and Clearfil Repair Kit experimental groups
Ozcan et al. (2013)	In vitro	*n* = 10	Zirconia (Lava Zirconia) and feldspathhic ceramic (VM7)	—	4% Hydrofluoric acid (90 s) on veneering ceramic + air abrasion with 50 μm aluminum oxide on zirconia (10 mm, 20 s, 2.8 bar), air abrasion with aluminum oxide on zirconia (with the veneering ceramic exposed) + hydrofluoric acid on veneering ceramic, hydrofluoric acid on veneering ceramic + air abrasion with 30 μm silica‐coated aluminum oxide on zirconia and air abrasion with silica‐coated aluminum oxide on zirconia (with the veneering ceramic exposed) + hydrofluoric acid on veneering ceramic	Silane (ESPE‐Sil) + adhesive resin (VisioBond)	Composite resin (Quadrant Posterior)	Bond strength (shear) and surface topography analysis (SEM)	Yes	The best bonding performance was obtained when the veneering ceramic was pre‐treated with hydrofluoric acid and the zirconia air abraded with silica coated aluminum oxide particles. Ideally, the veneer ceramic should be conditioned previously to the zirconia treatment
Özdemir and Yanikoglu (2017)	In vitro	*n* = 40	Feldspathic ceramic (VMK95 and IPS Classic)	Air abrasion with 50 μm aluminum oxide	9.5% Hydrofluoric acid + silane and air abrasion with 50 μm aluminum oxide + silane	—	Composite resin (Grandio or Ceram‐X)	Bond strength (shear) and surface topography analysis (SEM)	Yes	Hydrofluoric acid led to superior bond strength values in comparison to air abrasion. There were differences between the two types of feldspathic ceramic
Panah et al. (2008)	In vitro	*n* = 2	Lithium disilicate (IPS Empress 2)	No surface treatment	Air abrasion with 50 μm aluminum oxide (35 psi, 10 mm, 15 s), 9.6% hydrofluoric acid (30 s), silane, air abrasion + hydrofluoric acid, air abrasion + silane, hydrofluoric acid + silane, air abrasion + hydrofluoric acid + silane	Bonding resin (Heliobond)	Composite resin (Tetric Ceram)	Bond strength (shear)	No	The combination of air abrasion with aluminum oxide, hydrofluoric acid and silane rendered the highest repair bond strength values
Pedrazzi et al. (2012)	In vitro	*n* = 10	Feldspathic ceramic (Ceranco 3)	37% Phosphoric acid (15 s)	Laser (760 mW), laser (760 mW) + 37% OP acid (15 s), laser (900 mW), laser (900 mW) + 37% OP acid (15 s), 10% hydrofluoric acid (2 min)	Silane + adhesive system	Composite resin	Bond strength (shear) and surface topography analysis (SEM)	Yes	The use of Ti:sapphire laser rendered similar results to hydrofluoric acid, both superior to the control group where only phosphoric acid was used
Polat et al. (2021)	In vitro	*n* = 10	Zirconia (ICE Zirkon Translucent), feldspathic ceramic (Vita VMK) and zirconia with feldspathic ceramic	Diamond bur	Air abrasion with 50 μm aluminum oxide (4 bar, 20 s, 1 mm, 90°), Er,Cr:YSGG (2.78 μm, 10 mm, 200 μs, 20 Hz, 1.5 W), Er,Cr:YSGG (2.78 μm, 10 mm, 140 μs, 20 Hz, 1.5 W)	Clearfil Repair Kit (40% phosphoric acid was used only for the veneer ceramic)	Composite resin (Clearfil Majesty Esthetic)	Bond strength (shear) and surface topography analysis (SEM)	No	For zirconia, the control group (diamond bar) was the one where the highest values were achieved. With the combination of zirconia and veneer ceramic, both the laser and air abrasion treatment rendered similar results
Queiroz et al. (2012)	In vitro	*n* = 2	Feldspathic ceramic (Vita Mark II blocks)	—	Air abrasion with 30 μm silica‐coated aluminum oxide + silane, 10% hydrofluoric acid (60 s) + silane, hydrofluoric acid + Metal/Zirconia Primer, hydrofluoric acid + Clearfil Primer, hydrofluoric acid + Alloy Primer, hydrofluoric acid + V‐Primer, Metal/Zirconia Primer, Clearfil Primer, Alloy Primer, V‐Primer	Adhesive system (Scotchbond Multipurpose)	Composite resin (microhybrid, W3D Master, and nanofiller, Filtek Supreme)	Bond strength (microtensile)	Yes	The exclusive use of primers led to inferior bond strength values and mechanical pre‐treatments should be introduced in order to enhance the results
Rosa et al. (2024)	The present review summarized the scientific literature regarding protocols used for intraoral repairs of monolithic indirect restorations and observed that repairing with resin composite seems to be an interesting alternative for prolonging the longevity of indirect monolithic restorations									
Sadeghi et al. (2015)	In vitro	*n* = 12	Feldspathic ceramic (VITA Zahnfabrik)	No surface pretreatment	9% Hydrofluoric acid (90 s), Er:YAG laser (2 W, 100 mJ, 2.94 μm, 20 Hz, 20 s), Er:YAG laser (3 W, 150 mJ, 2.94 μm, 20 Hz, 20 s), Er:YAG laser (4 W, 200 mJ, 2.94 μm, 20 Hz, 20 s), Er:YAG laser (5 W, 250 mJ, 2.94 μm, 20 Hz, 20 s)	Silane (Ultradent) + adhesive (OptiBond XTR)	Composite resin (Point 4)	Bond strength (shear)	Yes	Hydrofluoric acid led to superior bond strength values than all experimental groups employing laser
Sagen et al. (2025)	In vitro	*n* = 10	Feldspathic ceramic (Initial Zr‐FS)	—	P220 rotating diamond disc and 9.5% hydrofluoric acid (90 s); universal adhesive and silane primer + adhesive	—	Composite resin (Filtek Supreme)	Bond strength (shear)	Yes	The separate silane primer and adhesive demonstrated superior bond strength over the universal adhesive.
Sanal and Kilinc (2020)	In vitro	*n* = 24	Feldspathic ceramic (VITA VM 9, VITA VM 13, VITA VMK 95) and leucite (IPS e.max Ceram)	—	Fusio Liquid Dentin (self‐adhesive flowable composite resin), Constic (self‐adhesive flowable composite resin), Vertise Flow (self‐adhesive flowable composite resin) and Bisco Repair Kit + Filtek Supreme (composite resin)	Diamond bur + air abrasion with 50 μm aluminum oxide (2 atm, 5 mm, 5 s) + hydrofluoric acid (60 s)	Composite resin (depending on the experimental group)	Bond strength (shear), color stability and surface topography analysis (SEM)	Yes	The evaluation of color stability showed unsatisfactory results for all the repair materials tested. The self‐adhesive flowable composite resins Fusio Liquid Dentin and Constic can be used as a replacement for the association of the repair kit and composite resin, for feldspathic ceramic.
Saraç et al. (2013)	In vitro	*n* = 15	Zirconia (ICE Zircon Translucent) and feldspathic ceramic (VITA Zahnfabrik)	No surface treatment	4% Hydrofluoric acid (5 min), Er:YAG laser (2 W, 200 mJ/pulse, 10 Hz, 10 s), CO_2_ laser (10.6 μm, 1000 Hz, 160 ms, 3 W), air abrasion with 50 μm aluminum oxide (10 mm, 2.5 bar, 15 s) and air abrasion with 30 μm silica‐coated aluminum oxide (10 mm, 20 s)	Silane primer (Porcelain Primer) + adhesive system (One‐Step Plus)	Composite resin (Reflexions)	Bond strength (shear) and surface topography analysis (SEM)	Yes	Air abrasion with silica‐coated aluminum oxide led to the highest bond strength values
Sattabanasuk et al. (2016)	In vitro	*n* = 10	Leucite (IPS Empress)	—	5% Hydrofluoric acid (60 s) and non‐etched; silane (RelyX) and not silanized; Adper Scotchbond Multi‐Purpose Adhesive and Scotchbond Universal	37% Phosphoric acid (15 s)	Composite resin (Filtek Z350)	Bond strength (shear), surface contact angle and surface topography analysis (SEM)	No	The application of silane as a separated step appears to be beneficial
Schellenber and Ozcan (2021)	In vitro	*n* = 10	Zirconia (IPS e.max ZirCAD), feldspathic ceramic (Creation), zirconia with feldspathic ceramic	Zirconia treated with air abrasion with 30 μm silica‐coated aluminum oxide (2.5 bar, 10 mm, 15 s) and feldspathic ceramic treated with 9.6% hydrofluoric acid (120 s) separately	Monobond Plus + adhesive resin (Heliobond), Clearfil Ceramic Primer Plus, Z prime Plus; both substrates treated with hydrofluoric acid + air abrasion + Monobond + Heliobond and both substrates treated with air abrasion + hydrofluoric acid + Monobond + Heliobond	—	Composite resin (Empress Esthetic Enamel)	Bond strength (shear)	Yes	From the three tested ceramic primers, Monobond Plus achieved the highest bond strength values. The sequence in which ceramic surface preparation was conducted did not influence the bond strength results
Seabra et al. (2014)	In vitro	*n* = 10	Zirconia (Lava Frame Zirconia)	—	Z‐Prime Plus (1 coat, without light‐curing), Z‐Prime Plus (1 coat, light‐cured), Z‐Prime Plus (2 coats, without light‐curing), Z‐Prime Plus (2 coats, light‐cured), All‐Bond Universal and ScotchBond Universal Adhesive	Air abrasion with 50 μm aluminum oxide (0.25 Mpa, 15 s, 10 mm)	Composite resin (Filtek Z250)	Bond strength (shear)	No	Regarding the zirconia primer, the best bond strength results for Z‐Prime Plus were obtained when two coats were applied followed by light‐curing. Both MDP‐containing adhesives (All‐Bond Universal and ScotchBond Universal Adhesive) rendered effective bond strength results
Shafiei et al. (2019)	In vitro	*n* = 10	Zirconia (Amann Girrbach)	No primer or resin	Alloy Primer, Z‐Prime Plus (without light polymerization), Z‐Prime Plus (with light polymerization), Alloy Primer + bonding resin (Clearfil SE Bond) and Z‐Prime Plus + bonding resin (Clearfil SE Bond)	Air abrasion with 35 μm aluminum oxide (2.5 bar, 10 mm, 15 s)	Composite resin (Clearfil APX)	Bond strength (shear)	Yes	Z‐Prime Plus combined with an additional resin layer provided the highest bond strength among the tested groups
Shoorgashti et al. (2024)	Hydrofluoric acid etching combined with silane application is the most effective method for enhancing bond strength in the repair of glass ceramic restorations									
Şişmanoğlu et al. (2020)	In vitro	*n* = 1	Feldspathic ceramic (Vitablocks Mark II)	No surface treatment	9% Hydrofluoric acid (60 s), air abrasion with 50 μm aluminum oxide (10 mm, 2.5 bar), air abrasion with 30 μm silica‐coated aluminum oxide (10 mm, 2.5 bar); Clearfil Ceramic Primer Plus or no silane coupling agent	Adhesive system (Single Bond Universal)	Composite resin (Filtek Ultimate Flowable Restorative)	Bond strength (shear), surface roughness analysis and surface topography analysis (SEM)	No	The association of a mechanical surface treatment and the application of silane rendered the highest bond strength values.
Soares et al. (2024)	In vitro	*n* = 15	Zirconia (IPS e.max ZirCAD)	Air abrasion with 50 μm aluminum oxide (2 bar, 10 mm, 10 s) + 10‐MDP primer	Air abrasion with 30 μm silica‐coated aluminum oxide (2 bar, 10 mm, 10 s) + MDP‐containing silane, air abrasion with 30 μm silica‐coated aluminum oxide (2 bar, 10 mm, 10 s) + MDP primer, universal adhesive system (Adhese Universal)	Diamond bur	Composite resin (Tetric EvoCeram)	Bond strength (shear), surface roughness analysis, flexural strength, finite element analysis and surface topography analysis (SEM)	Yes	The combination of mechanical and chemical approaches are crucial for ensuring durable bond strength and mechanical stability in zirconia repairs.
Soares et al. (2024)	In vitro	*n* = 15	Zirconia (IPS e.max ZirCAD)	—	Nanohybrid resin composite (Tetric Evo Ceram), bulk‐fill resin composite (Tetric PowerFill), and flowable resin (Tetric PowerFlow)	Diamond bur + air abrasion with 50 μm aluminum oxide (10 mm, 2.8 bar, 10 s) + 10‐MDP primer (Alloy primer)	Composite resin (Tetric Evo Ceram, Tetric PowerFill) and flowable composite resin (Tetric PowerFlow)	Bond strength (shear), flexural fatigue strength and finite element analysis	Yes	The different types of composite resin did not achieve statistically distinct results
Soares et al. (2024)	In vitro	*n* = 6	Lithium disilicate (IPS e.max) and composite block (Tetric CAD)	—	Brushing movement parallel to the bonding interface of the repair and perpendicular to the bonding interface of the repair	Lithium disilicate: primer (Monobond Etch & Prime)	Composite resin (Clearfil AP‐X)	Surface roughness analysis and surface topography analysis (SEM)	No	Brushing simulation influenced the surface roughness of ceramic restorations compared with composite resin
Suárez‐Moya et al. (2019)	In vitro	*n* = 10	Feldspathic ceramic (Vitablocs TriLuxe)	—	Single Bond Universal, Adper Single Bond 2, silane + Single Bond Universal, silane + Adper Single Bond 2, 9.6% hydrofluoric acid (90 s) + silane + Single Bond Universal, 9.6% hydrofluoric acid (90 s) + silane + Adper Single Bond 2	Air abrasion with 50 μm aluminum oxide (10 mm, 2.5 bar, 90°, 10 s)	Composite resin (Filtek Z350)	Bond strength (shear)	No	Both adhesive systems rendered similar bond strength values but the independent application of silane is crucial to enhance bonding performance
Tarcin et al. (2011)	In vitro	*n* = 20	Leucite (Vitablocs)	No surface mechanical treatment with both adhesive systems	Er:YAG laser (2.94 μm, 10 Hz, 200 mJ, 300 μs, 2 W, 20 s) and air abrasion with 50 μm aluminum oxide (2.5 bar, 10 mm, 15 s, 90°); 36% OP acid + One‐Step Plus + silane (Monobond) and All‐Bond SE + silane (Monobond)	—	Composite resin (Aelite Flo LV)	Bond strength (shear)	No	The combination of chemical and mechanical surface treatments led to the highest bond strength values
Tavares et al. (2024)	In vitro	*n* = 11	Lithium disilicate (IPS e.max press)	No surface treatment	Three different designs of the top surface (flat, single 2‐mm‐deep plateau, or double 2‐mm‐deep plateau); 5% hydrofluoric acid (20 s) and air abrasion with 50 μm aluminum oxide (5 mm, 10 s)	Silane (Rely X Ceramic Primer) + adhesive system (Single Bond Universal)	Composite resin (Filtek Z350)	Bond strength (microtensile) and surface topography analysis (SEM)	Yes	The experimental groups pre‐treated with hydrofluoric acid presented higher bond strength values than the samples treated with air abrasion.
Tokar et al. (2019)	In vitro	*n* = 10	Zirconia (ICE Zirkon Translucent) and feldspathic ceramic (Vita VMK Master)	Diamond bur	Er,Cr:YSGG laser (2.78 mm, 20 Hz, 0.25 mm, 6.0 W): pulse duration was set 140 ms for short‐pulse and 200 ms for long‐pulse	Ceramic Repair N (Monobond + Heliobond)	Composite resin (Tetric N‐Ceram)	Bond strength (shear) and surface topography analysis (SEM)	No	Although not statistically significant, short pulse laser irradiation achieved superior results in comparison to long pulse rate. The control group was associated with the highest bond strength values
Turunç‐Oğuzman and Şişmanoğlu (2023)	In vitro	*n* = 12	Feldspathic (Vita Mark II) and lithium disilicate (IPS e.max)	No surface treatment, with adhesive system or silane + adhesive system	Air abrasion with 50 μm aluminum oxide (10 mm, 1.5 bar), 9% hydrofluoric acid (60 s) and Monobond Etch and Prime; Clearfil Ceramic Primer Plus + adhesive system (Heliobond) and adhesive system (Single Bond Universal)	—	Composite resin (Filtek Ultimate Flowable)	Bond strength (shear) and surface topography analysis (SEM)	No	For lithium disilicate, the best bonding performance was achieved with Monobond Etch and Prime + conventional adhesive (Heliobond). For the feldspathic ceramic, the use of hydrofluoric acid rendered the best bond strength results
Ueda et al. (2021)	In vitro	*n* = 6	Lithium disilicate (IPS e.max)	No surface treatment (only for surface roughness analysis)	Monobond Etch & Prime, K‐etchant GEL + Clearfil Universal Bond, Bondmer Lightless, K‐etchant GEL + G‐Multi PRIMER	—	Composite resin (Clearfil AP‐X)	Bond strength (shear), surface roughness and surface topography analysis (SEM)	Yes	Monobond Etch & Prime was associated with the highest bond strength results
Üstün et al. (2018)	In vitro	*n* = 10	Lithium disilicate (IPS e.max) and zirconia‐reinforced lithium disilicate ceramic (Vita Suprinity)	—	IPS e.max and Vita Suprinity: 9% hydrofluoric acid (20 s); Ceramic Repair (37% phosphoric acid (5 s) + Monobond S + Heliobond) and 40% phosphoric acid + Clearfil SE Bond Primer + Clearfil Porcelain Bond Activator + Clearfil SE Bond	—	Composite resin (Tetric N Ceram or Filtek Z250)	Bond strength (shear) and surface topography analysis (SEM)	Yes	For lithium disilicate ceramic, Clearfil Repair achieved superior bond strength results than Ceramic Repair.
Uzun et al. (2015)	In vitro	*n* = 10	Zirconia (ICE Zirkon) and feldspathic ceramic (Cerabien Zr)	Bur	Air abrasion with 50 μm aluminum oxide (2.8 bar, 10 mm, 15 s) and Er,Cr:YSGG laser (2940 nm, 400 μm, 2 W, 10 s)	Silane (Cimara Zircon‐Primer) + adhesive (Cimara Zircon‐Adhesive)	Composite resin (Grandio SO)	Bond strength (shear) and surface topography analysis (SEM)	no	The control group (bur) was associated with the highest bond strength values, for both types of ceramic
Valian et al. (2021)	In vitro	*n* = 10	Feldspathic ceramic (Ceramco 3)	Two‐part silane (Bis‐Silane) + porcelain bonding resin (Porcelain Bond)	Single Bond Universal adhesive, silane (Bis‐silane) + Single Bond Universal adhesive, All Bond Universal bonding agent, silane (Bis‐silane) + All Bond Universal bonding agent, FuturaBond Universal bonding agent and silane (Bis‐silane) + FuturaBond Universal bonding agent	9.5% Hydrofluoric acid (2 min)	Composite resin (Filtek Z250)	Bond strength (shear)	Yes	The use of hydrofluoric acid should be associated with the separated application of silane
Veríssimo et al. (2020)	In vitro	*n* = 10	Zirconia‐reinforced lithium disilicate ceramic (Vita Suprinity)	No surface treatment	Diamond bur + adhesive system (Single Bond Universal), diamond bur + silane + adhesive system (Amber), 10% hydrofluoric acid (20 s) + silane + adhesive system (Amber), air abrasion with 30 μm silica‐coated aluminum oxide (20 s, 15 mm, 2.5 bar) + silane + adhesive system (Amber), air abrasion with aluminum oxide (20 s, 15 mm, 2.5 bar) + silane + adhesive system (Amber)	—	Composite resin (Filtek Z350)	Bond strength (shear), fungal and bacterial colony forming units, EDS analysis, surface roughness analysis and surface topography analysis (SEM)	Yes	Acid etching followed by silanization rendered the highest bond strength values for zirconia‐reinforced lithium disilicate ceramic
Yildirim et al. (2019)	In vitro	*n* = 12	Feldspathic ceramic (Vitablocks Mark II)	—	5% Hydrofluoric acid (60 s) + silane (Monobond Plus), Monobond Etch and Prime, air abrasion with 50 μm aluminum oxide (5 s, 10 mm, 0.5 bar) + silane (Monobond Plus) and air abrasion + Monobond Etch and Prime	Bonding resin (Heliobond)	Composite resin (Tetric Evo Ceram)	Bond strength (shear) and surface topography analysis (SEM)	Yes	The experimental group with hydrofluoric acid treatment rendered the highest bond strength values
Zaghloul et al. (2014)	In vitro	*n* = 2	Leucite (Paradigm C) and resin composite block (Paradgim MZ100)	—	Diamond bur + 30% phosphoric acid (30 s), diamond bur + 30% phosphoric acid (30 s) + silane (RelyX Ceramic Primer), 3% hydrofluoric acid (90 s), 3% hydrofluoric acid (90 s) + silane, air abrasion with 30 μm silica‐coated aluminum oxide (2.5 bar, 10 s, 10 mm), air abrasion + silane	Adhesive system (Single Bond Universal)	Composite resin (Filtek Z350)	Bond strength (shear) and surface topography analysis (SEM)	No	The association of hydrofluoric acid and silane led to the highest bond strength values

Out of the total 126 publications considered, 7 papers are reviews [1, 2, 4, 5, 14, 15, 16] while the others are in vitro studies [7, 8, 9, 10, 11, 17, 18, 19, 20, 21, 22, 23, 24, 25, 26, 27, 28, 29, 30, 31, 32, 33, 34, 35, 36, 37, 38, 39, 40, 41, 42, 43, 44, 45, 46, 47, 48, 49, 50, 51, 52, 53, 54, 55, 56, 57, 58, 59, 60, 61, 62, 63, 64, 65, 66, 67, 68, 69, 70, 71, 72, 73, 74, 75, 76, 77, 78, 79, 80, 81, 82, 83, 84, 85, 86, 87, 88, 89, 90, 91, 92, 93, 94, 95, 96, 97, 98, 99, 100, 101, 102, 103, 104, 105, 106, 107, 108, 109, 110, 111, 112, 113, 114, 115, 116, 117, 118, 119, 120, 121, 122, 123, 124, 125, 126, 127, 128, 129, 130, 131]. The considered ceramics included glass ceramics [7, 9, 11, 17, 18, 19, 20, 21, 22, 23, 24, 25, 26, 27, 28, 29, 30, 31, 32, 33, 34, 35, 36, 37, 38, 39, 40, 41, 42, 43, 44, 45, 46, 47, 48, 49, 50, 51, 52, 53, 54, 55, 56, 57, 58, 59, 60, 61, 62, 63, 64, 65, 66, 67, 68, 69, 70, 71, 72, 73, 74, 75, 76, 77, 78, 79, 80, 81, 82, 83, 84, 85, 86, 87, 88, 89, 90, 91, 92, 93, 94, 95, 130, 131] and metal oxide ceramics [7, 8, 9, 10, 11, 18, 22, 35, 43, 44, 48, 50, 53, 56, 59, 62, 67, 84, 90, 92, 93, 96, 97, 98, 99, 100, 101, 102, 103, 104, 105, 106, 107, 108, 109, 110, 111, 112, 113, 114, 115, 116, 117, 118, 131]. The absence of surface treatment was the most used control among publications [8, 9, 10, 11, 19, 23, 24, 27, 31, 34, 37, 48, 54, 63, 67, 70, 73, 78, 80, 82, 91, 93, 100, 108, 109, 110, 115, 118, 120, 121, 122, 125, 126, 129]. Composite resin was used as the repair material in 97.5% of the included papers [7, 8, 9, 10, 11, 17, 18, 19, 20, 21, 22, 23, 24, 25, 26, 27, 28, 29, 30, 31, 32, 33, 34, 35, 36, 37, 38, 39, 40, 41, 42, 43, 44, 45, 46, 47, 48, 49, 50, 51, 52, 53, 54, 55, 56, 57, 58, 59, 60, 62, 64, 65, 66, 67, 68, 69, 70, 71, 72, 73, 74, 75, 76, 77, 78, 79, 80, 81, 82, 83, 84, 85, 86, 87, 88, 89, 90, 91, 92, 93, 94, 95, 96, 97, 98, 99, 100, 101, 102, 103, 105, 106, 107, 108, 109, 110, 111, 112, 113, 114, 115, 116, 119, 121, 122, 123, 124, 125, 126, 127, 128, 129, 130, 131], while ceramic was employed in 2.5% [39, 63, 86, 120]. Bond strength was the most frequently evaluated outcome among the included publications [7, 8, 9, 10, 11, 17, 18, 19, 20, 21, 22, 23, 24, 25, 26, 27, 28, 29, 30, 31, 32, 33, 34, 35, 36, 37, 38, 39, 40, 41, 42, 43, 44, 45, 46, 47, 48, 50, 51, 52, 54, 55, 56, 57, 59, 60, 61, 62, 63, 64, 65, 66, 67, 68, 69, 70, 71, 72, 73, 74, 75, 76, 77, 78, 80, 81, 82, 83, 84, 85, 88, 89, 90, 91, 92, 93, 94, 95, 96, 97, 98, 99, 100, 101, 102, 103, 104, 105, 106, 107, 108, 109, 110, 111, 112, 113, 114, 115, 116, 117, 118, 119, 120, 121, 122, 123, 125, 126, 127, 128, 129, 130], followed by ultrastructural analysis [10, 18, 22, 23, 24, 25, 26, 28, 30, 31, 32, 33, 34, 37, 38, 39, 40, 43, 46, 47, 50, 52, 57, 59, 61, 62, 64, 65, 66, 67, 70, 72, 75, 78, 79, 81, 83, 86, 88, 91, 94, 97, 98, 99, 100, 101, 103, 104, 106, 109, 110, 111, 114, 116, 118, 119, 123, 126, 129, 130], chemical analysis [23, 38, 88, 109, 110, 126], color evaluation [20, 49, 58, 61, 87, 131], and mechanical properties [53, 86, 98, 111]. Long‐term evaluation resorting to aging methods was conducted in 63.9% of the papers [7, 8, 9, 11, 17, 21, 23, 25, 26, 27, 28, 29, 30, 32, 33, 34, 35, 39, 40, 41, 42, 43, 44, 46, 48, 53, 54, 55, 56, 58, 60, 61, 62, 63, 65, 66, 67, 71, 72, 74, 76, 77, 78, 81, 84, 85, 86, 87, 88, 89, 90, 94, 95, 96, 97, 98, 101, 103, 105, 107, 109, 110, 111, 112, 113, 114, 115, 118, 120, 121, 125, 126, 127, 129, 131].

## 4 Discussion

Ceramic type, repair material, surface treatment, and aging process are parameters that can influence the evaluation of repair procedures. Several types of ceramics were tested among the included studies, with the majority focusing on feldspathic ceramics [7, 9, 11, 17, 18, 21, 22, 26, 27, 28, 29, 31, 32, 34, 35, 36, 38, 42, 43, 44, 46, 48, 50, 51, 52, 53, 55, 56, 57, 59, 60, 61, 63, 65, 67, 70, 71, 72, 74, 76, 77, 81, 82, 83, 84, 85, 86, 88, 93, 95, 117, 121, 128, 129], followed by zirconia [7, 8, 9, 10, 11, 18, 22, 35, 43, 44, 48, 50, 53, 56, 59, 62, 67, 84, 90, 92, 93, 96, 97, 98, 99, 100, 101, 102, 103, 104, 105, 106, 107, 108, 109, 110, 111, 112, 113, 114, 115, 116, 117, 118, 122, 131], lithium disilicate [18, 19, 21, 23, 25, 27, 30, 31, 33, 36, 37, 40, 41, 43, 52, 54, 62, 65, 66, 69, 73, 78, 79, 89, 90, 92, 94, 120, 125, 131], leucite [20, 24, 27, 30, 39, 41, 43, 45, 47, 49, 58, 61, 64, 65, 68, 73, 75, 80, 87, 91, 130], zirconia‐reinforced lithium silicate ceramics [8, 9, 33, 43, 54, 57, 62, 119, 123, 126, 127], alumina [18, 56, 117], and lithium aluminosilicate ceramics reinforced with lithium disilicate [8]. Substrate identification is a crucial step in establishing a ceramic repair protocol; therefore, literature interpretation should always consider the type of ceramic involved.

The vast majority of the in vitro studies included in this review employed composite resin as the repair material, either in a flowable or medium consistency form. Studies comparing different composite resins reported no statistically significant differences in bond strength values [29, 55, 98]. Furthermore, flexural fatigue was also not influenced by the type of composite resin employed [98]. Apart from composite resin, ceramic was also tested as a repair material resulting in enhanced bond strength values compared to composite resin [39, 85, 86, 110]. In addition, it is noteworthy that repair with lithium disilicate rendered higher bond strength values than feldspathic ceramic [110], and the former was also associated with superior fracture resistance [86].

### 4.1 Bond Strength

Bond strength was the most frequently evaluated outcome among the included publications, assessed either by shear [7, 8, 10, 11, 17, 19, 20, 22, 23, 24, 25, 26, 28, 29, 30, 31, 32, 33, 34, 35, 36, 37, 38, 40, 41, 42, 43, 44, 48, 50, 51, 52, 56, 59, 60, 61, 62, 63, 64, 65, 67, 68, 70, 71, 72, 74, 75, 76, 80, 82, 83, 84, 85, 88, 89, 90, 92, 94, 95, 96, 97, 98, 99, 100, 101, 102, 103, 105, 106, 107, 108, 109, 110, 111, 112, 113, 114, 115, 116, 117, 118, 121, 122, 123, 125, 126, 128, 130] or microtensile [9, 18, 21, 27, 39, 45, 46, 47, 54, 55, 57, 66, 69, 73, 77, 78, 81, 91, 93, 104, 119, 120, 127, 129] testing. Although 77.7% of the studies resorted to shear testing, this method can be associated with inconsistent and heterogeneous stress distribution, consequently influencing the interpretation of results [2, 54, 55, 91, 105].

Regarding glass ceramics, hydrofluoric acid has been widely recognized as the most effective surface conditioning treatment [8, 21, 31, 37, 125, 129], due to its ability to dissolve the glass phase, expose the crystalline matrix and enhance micromechanical retention [1, 9, 31, 34, 37, 43, 45, 60, 69, 83, 114, 128]. Over the years, studies have shown a tendency to use lower hydrofluoric acid concentrations and shorter etching times [1]. It is noteworthy that, in contrast to hydrofluoric acid, phosphoric acid is unable to interact effectively with glass ceramics [23, 30, 66].

Although literature recognizes the role of hydrofluoric acid as an effective ceramic surface treatment, some of the included articles express concerns regarding its potential harmful effects upon contact with soft tissues during intraoral repair of ceramic restorations [9, 34, 120]. Taking into account the associated risks, the use of absolute isolation under rubber dam is mandatory [4, 27, 125], as bond strength values are also exponentiated under these conditions [120, 132]. In addition, it is noteworthy that alternative pretreatments, such as air abrasion, also require rubber dam isolation [27, 133].

Hydrofluoric acid was associated with higher repair bond strength values than air abrasion for all glass ceramics [21, 26, 31, 37, 52, 60, 63, 72, 93, 119, 128]. Regarding the abrasive particles, silica‐coated aluminum oxide increases the silica content on the ceramic surface, favoring subsequent interactions with silane [27, 47], while residual aluminum oxide particles may hinder the interaction with bonding agents [120]. Although some reports indicate that air abrasion yields similar results to hydrofluoric acid treatment [47], this pretreatment only leads to the highest bond strength values among experimental groups when direct comparison with hydrofluoric acid is not performed and, therefore, is considered insufficient to achieve optimal bonding on its own [30].

As an alternative to the effect of hydrofluoric acid or air abrasion on glass ceramics, different authors tested the influence of laser treatment [8, 11, 22, 23, 31, 32, 34, 35, 37, 38, 40, 43, 46, 48, 50, 59, 62, 65, 67, 68, 71, 80, 88, 90, 121, 123, 125]. In the majority of the included publications, laser irradiation rendered inferior bond strength results compared to hydrofluoric acid [37, 40, 43, 62, 67, 90, 92, 121, 125], but comparisons with air abrasion were contradictory [35, 65, 67]. As there is no clear consensus regarding the optimal power settings for laser irradiation [23], some reports indicate that excessive heat generation can cause significant damage to the outer ceramic layer, thereby hindering the subsequent bonding process [40].

Other alternative surface treatments have been tested, such as atmospheric‐pressure plasma and UV irradiation, but neither has achieved results comparable to those obtained with hydrofluoric acid [45].

In addition to mechanical pretreatments, silane is to increase surface energy and wettability [8, 16, 23, 26, 54, 89]. Its interaction with the ceramic crystalline phase and the organic matrix of the resin allows for the creation of a strong bonding interface [34, 83]. Literature is consensual in stating that the sole use of a universal adhesive containing silane cannot be equated to a separate silanization step, which has been proven to be more effective [51, 64, 70, 74, 89, 95]. This can be explained by the inactivation of the condensation reaction induced by silane when different components are mixed together within the same bottle [89]. Although the use of a bonding agent is believed to be advantageous [7, 128], available literature fails to provide comparable bond strength results with or without the application of a bonding resin.

Overall, the combination of hydrofluoric acid etching and silanization provided the strongest bond between composite resin and glass ceramics [5, 39, 40, 62, 64, 70, 78, 90]. The concomitant use of air abrasion in addition to this combination can further potentiate bonding interactions [18, 19, 60]. Some of the included studies reported results regarding a self‐etching ceramic primer, Monobond Etch & Prime (Ivoclar Vivadent, Schaan, Liechtenstein) [25, 52, 72, 79, 94, 120], which attempts to simplify the repair protocol by combining etching and silanization steps. Although some studies reported bond strength results superior to air abrasion [120] but similar [94] or inferior [72] to hydrofluoric acid, the literature lacks more direct comparisons with the latter.

Apart from conventional glass ceramics, publications evaluating zirconia‐reinforced lithium disilicate ceramics [8, 9, 33, 43, 54, 57, 62, 119, 123, 126, 127] or lithium aluminosilicate ceramics reinforced with lithium disilicate [8] demonstrated these materials' sensitivity to hydrofluoric acid [8, 62], as a consequence of their elevated silica content. Therefore, acid etching followed by silanization rendered the highest bond strength values [126]. It is noteworthy that, for both materials, laser irradiation did not have a significant impact [8].

In comparison to glass ceramics, silica‐free ceramics, such as zirconia and alumina, are less susceptible to etching [2, 4, 9, 11]. For this reason, hydrofluoric acid does not have the ability to increase surface roughness [9]. As a consequence, air abrasion is more efficient for the repair of metal oxide ceramics [62], significantly improving the bond strength between ceramic and composite resin [103].

The use of silica‐coated aluminum oxide particles led to better results compared to aluminum oxide particles alone [18, 35, 107, 108, 112]. This can be explained by the fact that the increased silica content on the surface promotes stronger chemical interaction with the subsequently applied bonding agent [35, 108, 110, 112]. Despite this, it is noteworthy that, because silica remains superficially attached rather than chemically bonded to the ceramic, it can dislodge over time, compromising the long‐term interaction [109, 110].

Air abrasion results in superior bond strength values compared to laser treatment [10, 35, 90], with studies reporting either statistically similar [10] or superior [11] surface roughness for air abrasion, leading to enhanced wettability and mechanical interlocking [11]. In addition, the combination of laser and air abrasion has been reported to potentiate bond strength for metal oxide ceramic repairs [122]. Nevertheless, laser treatment is more effective in zirconia repair than in glass ceramics [35].

Mechanical pretreatments have been shown to improve bonding performance by creating microretentions; however, this alone is insufficient to achieve clinically acceptable bond strength values [102]. Silane coupling agents are less effective on zirconia than on glass ceramics due to the lower silica content [35, 68, 106]. Conversely, to achieve a proper chemical bond to zirconia, primers containing MDP (10‐methacryloyloxydecyl dihydrogen phosphate) monomer should be used [11, 35, 103, 104, 106, 118], as they promote interaction between the oxidic substrate and the composite resin [112].

In addition to the studies that conducted the repair protocol on a homogeneous surface, seven articles evaluated the repair bond strength for hybrid substrates [7, 11, 43, 44, 59, 84, 117], simulating a clinical scenario in which the fracture exposes more than one material. While the majority focused on the simultaneous exposure of zirconia and feldspathic ceramics [2, 6, 43, 66, 106, 119], combinations such as zirconia/leucite [43], alumina/feldspathic [117], and lithium disilicate/leucite [43] were also reported. Most of the included studies employed the same treatment strategy for both exposed substrates [11, 43, 59, 117]. Furthermore, in this scenario, most articles avoided the use of hydrofluoric acid [11, 44, 59, 117].

Studies that adapted the surface treatment to the exposed substrate, conditioned zirconia with air abrasion with silica‐coated aluminum oxide and feldspathic ceramic with hydrofluoric acid [7, 84]. Although one paper reported that the sequence of ceramic surface preparation did not influence bond strength results [84], another demonstrated that, ideally, the glass ceramic should be conditioned before zirconia treatment [7]. It is noteworthy that, although the materials were prepared individually, the authors do not mention any isolation method to prevent cross‐contamination, which may be a fundamental consideration for intra‐oral workflows.

### 4.2 Ultrastructural Analysis

In addition to bond strength tests, the majority of studies conducted surface topography analysis through scanning electron microscopy (SEM) [18, 22, 23, 24, 25, 26, 28, 30, 31, 32, 33, 34, 38, 40, 43, 46, 47, 50, 52, 59, 61, 62, 64, 65, 66, 67, 70, 72, 75, 78, 79, 81, 83, 86, 91, 94, 97, 99, 100, 101, 103, 109, 110, 111, 114, 116, 118, 119, 123, 126, 129, 130], field‐emission scanning electron microscopy (FE‐SEM) [37, 106], atomic force microscopy (AFM) [57], and surface roughness measurements [10, 25, 30, 38, 39, 70, 79, 88, 104, 106, 109, 110, 111, 114, 123, 126, 129, 130].

The evaluation of surface topography demonstrated the induction of deep surface irregularities in glass ceramics treated with hydrofluoric acid [28, 34, 43]; however, this effect was not observed in zirconia [62]. For glass ceramics, air abrasion can lead to the formation and propagation of cracks [126], and fewer microporosities are created compared to the hydrofluoric acid treatment [37, 43, 62]. On the other hand, air abrasion is able to induce significant surface modifications in zirconia [99, 100, 109, 110]. Self‐etching ceramic primers are also associated with higher surface roughness than untreated ceramic surfaces; consequently, increasing micromechanical retention [25].

Furthermore, laser treatment induces surface modifications characterized by homogenous, shallow and evenly distributed roughness, with no associated cracks or fissures [22, 34, 38]; however, it is considered insufficient to influence bond strength results compared to hydrofluoric acid [37, 43, 46, 62] and air abrasion [43, 62]. Higher laser power settings lead to added surface irregularities [125], with Nd:YAG lasers producing more pronounced alterations than Er:YAG lasers [34].

Publications have reported enhanced wettability associated with surface treatments that induce greater surface alterations [30, 45, 56, 106]. Although surface topography analysis should be interpreted alongside bond strength evaluation, it is important to keep in mind that surface structural modifications are not the sole factor influencing bond strength outcomes [37].

### 4.3 Chemical Analysis

Six studies included chemical analysis of the treated ceramics, performed using either energy dispersive spectroscopy (EDS) [23, 38, 109, 110, 126], Fourier‐transform infrared spectroscopy (FTIR) [38], or Raman spectroscopy [88].

Some studies report that repair protocols have minimal effect on the material's overall composition [126] while others demonstrate significant differences, although a correlation with other outcomes, such as bond strength results, cannot always be established [23]. EDS analysis of feldspathic ceramics revealed that surface treatment with hydrofluoric acid is strongly associated with chemical alterations, with a reduction in the concentrations of Si, K, Al, and Na, along with the introduction of new elements. In addition, conditioning of zirconia‐reinforced lithium disilicate showed a more consistent elemental composition across groups, as the dissolution of the silica matrix and consequent exposure of zirconia particles caused by hydrofluoric acid was not sufficient to alter the chemical content [126].

Elraggal et al. compared the use of air abrasion with fluorapatite glass–ceramic powder and silica‐coated aluminum oxide on zirconia [109, 110]. EDS analysis demonstrated a higher silica content after air abrasion with fluorapatite glass–ceramic powder compared to silica‐coated aluminum oxide [109, 110], which was also associated with superior bond strength values for that experimental group [109].

The remaining studies reporting chemical analysis evaluated the effect of carbon dioxide laser treatment on feldspathic ceramics, with FTIR spectra confirming good interaction between laser and the ceramic substrate [38], and Raman spectroscopy revealing that the reduction of isolated Si–OH groups caused by laser irradiation potentiates the interaction with silane [88]. This result was also associated with superior bond strength [88].

### 4.4 Color

Color evaluation was reported in six publications [20, 49, 58, 61, 87, 131]. The aging process influenced the translucency of the restorations, leading to a mismatch between the ceramic and the composite resin used for repair [87]. Translucency and color differences also varied under different illuminants [87].

As color stability is directly associated with water sorption and the hydrophilicity of repair materials, Sanal et al. assessed the color stability of ceramic repairs with four different composite resins, using coffee as a staining agent. All experimental groups presented unsatisfactory results, with Δ*E* exceeding the acceptability thresholds [61]. Furthermore, Kim et al. assessed the metameric color differences between various leucite shades and several composite resins. Metameric effects varied depending on porcelain shade, resin composite brand, and illuminant, with interactions observed between these factors [49].

Air abrasion with aluminum oxide, silica‐coated aluminum oxide and bioactive glass did not interfere with the color stability of leucite ceramics [58]. It is noteworthy that this study did not mention the use of hydrofluoric acid. In addition, two universal shade composites were compared after different surface preparations, with Essentia (GC Corp., Tokyo, Japan) presenting better color stability than Omnichroma (Tokuyama Dental Corp., Tokyo, Japan) [58].

Although color stability is recognized as a key factor in the success of repaired restorations, it should be taken into account that few studies address strategies to minimize optical differences between substrates, such as creating a bevel [21, 97] in the ceramic substrate before the repair procedure.

### 4.5 Mechanical Properties

Four publications assessed the mechanical properties of repaired ceramic restorations, either through flexural strength [98, 111] or fracture resistance [53, 86] evaluation.

Both studies evaluating flexural strength in repaired zirconia [98, 111] concluded that surface treatments can influence mechanical behavior, with air abrasion with silica‐coated aluminum oxide and an MDP‐containing silane achieving not only the highest bond strength values, but also the highest flexural strength values [111]. The authors hypothesized that the combination of mechanical and chemical treatments reinforces the repaired restoration and, therefore, this association is crucial for optimal zirconia repair [111]. In contrast, the use of different types of composite resins did not result in statistically significant differences in flexural strength outcomes [98].

Regarding fracture resistance, zirconia treatment with air abrasion using silica‐coated aluminum oxide particles and silane rendered the highest results compared to treatment with a bur or conventional aluminum oxide air abrasion [53]. It is noteworthy that cyclic loading diminished the fracture resistance of all repaired zirconia crowns, regardless of the pretreatment employed [53]. The other study assessing fracture resistance evaluated veneered zirconia repaired with either composite resin or ceramic [86]. Ceramic repair rendered superior fracture resistance compared to composite resin, with lithium disilicate performing better than feldspathic ceramic [86].

## 5 Limitations

The primary limitation of this review is the substantial methodological heterogeneity among the included studies and the total absence of randomized clinical trials on this topic. In addition, 3 publications failed to report the sample size, and only 19 studies included sample size calculation [31, 35, 41, 52, 58, 63, 72, 91, 92, 93, 98, 102, 108, 111, 115, 118, 126, 129, 130]. When reported, sample size ranged from 1 to 72, although it should be taken into account that some authors considered an entire ceramic block, while others considered only a disk. Furthermore, it should be noted that a wide variety of outcomes were evaluated, and that many studies lack adequate control groups or did not align with authentic clinical conditions, with papers reporting the use of ultrasonic cleaning as a fractured ceramic pretreatment before the repair protocol, which cannot be performed in an actual clinical scenario. Another major limitation is the reduced number of publications reporting repair protocols on hybrid substrates requiring distinct pretreatments, which would more accurately reflect the clinical complexity and inherent risk of cross‐contamination.

## 6 Conclusion

Based on this scoping review, the following conclusions can be drawn:

- Methodological heterogeneity among studies and the absence of clinical trials precludes the establishment of definitive clinical conclusions;
- In ceramic repair procedures, glass ceramics should be treated with a combination of hydrofluoric acid etching and silanization, while metal oxide ceramics demonstrate superior outcomes when air abrasion and an MDP‐containing primer are employed;
- Standardized methodologies should be established to enable the design of more consistent and comparable laboratory and clinical studies.

## Funding

The authors have nothing to report.

## Disclosure

The authors have nothing to report.

## Conflicts of Interest

The authors declare no conflicts of interest.

## Supporting information


**Data S1:** Supporting Information.

## Data Availability

The data that support the findings of this study are available from the corresponding author upon reasonable request.

## References

[1] I. O. Nogueira , C. N. B. Pereira , L. G. Abreu , I. M. A. Diniz , C. S. Magalhães , and R. R. D. Silveira , “Do Different Protocols Affect the Success Rate or Bond Strength of Glass‐Ceramics Repaired With Composite Resin? A Systematic Review and Meta‐Analysis,” Journal of Prosthetic Dentistry 133, no. 5 (2025): 1157–1171. 37635007 10.1016/j.prosdent.2023.06.020

[2] A. Della‐Bona , “Characterizing Ceramics and the Interfacial Adhesion to Resin: II—The Relationship of Surface Treatment, Bond Strength, Interfacial Toughness and Fractography,” Journal of Applied Oral Science 13, no. 2 (2005): 101–109. 20924531 10.1590/s1678-77572005000200002

[3] A. Muhetaer , C. Tang , A. Anniwaer , H. Yang , and C. Huang , “Advances in Ceramics for Tooth Repair: From Bench to Chairside,” Journal of Dentistry 146 (2024): 105053. 38729288 10.1016/j.jdent.2024.105053

[4] L. S. da Rosa , R. O. Pilecco , P. M. Soares , et al., “Repair Protocols for Indirect Monolithic Restorations: A Literature Review,” PeerJ 12 (2024): e16942. 38406292 10.7717/peerj.16942 PMC10893862

[5] R. Shoorgashti , S. S. Ehsani , M. Ducret , and R. Rokhshad , “Effect of Surface Treatments on the Bond Strength of Computer‐Aided Design and Computer‐Aided Manufacturing Lithium Disilicate to Restorative Materials: A Systematic Review,” European Journal of Prosthodontics and Restorative Dentistry 32, no. 4 (2024): 423–433. 39607319 10.1922/EJPRD_2777Shoorgashi11

[6] FDI World Dental Federation , “Repair of Restorations: Adopted by the General Assembly: September 2019, San Francisco, United States of America,” International Dental Journal 70, no. 1 (2020): 7–8. 31985815 10.1111/idj.12552 PMC9379206

[7] M. Ozcan , L. F. Valandro , S. M. Pereira , R. Amaral , M. A. Bottino , and G. Pekkan , “Effect of Surface Conditioning Modalities on the Repair Bond Strength of Resin Composite to the Zirconia Core/Veneering Ceramic Complex,” Journal of Adhesive Dentistry 15, no. 3 (2013): 207–210. 23700578 10.3290/j.jad.a29717

[8] S. Ü. Aladağ and E. A. Ayaz , “Repair Bond Strength of Different CAD‐CAM Ceramics After Various Surface Treatments Combined With Laser Irradiation,” Lasers in Medical Science 38, no. 1 (2023): 51. 36689017 10.1007/s10103-023-03715-3

[9] L. Al‐Turki , Y. Merdad , T. A. Abuhaimed , D. Sabbahi , M. Almarshadi , and R. Aldabbagh , “Repair Bond Strength of Dental Computer‐Aided Design/Computer‐Aided Manufactured Ceramics After Different Surface Treatments,” Journal of Esthetic and Restorative Dentistry 32, no. 7 (2020): 726–733. 32886852 10.1111/jerd.12635

[10] S. Arami , M. H. Tabatabaei , F. Namdar , N. Safavi , and N. Chiniforush , “Shear Bond Strength of the Repair Composite Resin to Zirconia Ceramic by Different Surface Treatment,” Journal of Lasers in Medical Sciences 5, no. 4 (2014): 171–175. 25653817 PMC4281985

[11] M. A. Abdulla and R. H. Hasan , “Shear Bond Strength of Two Repair Systems to Zirconia Ceramic by Different Surface Treatments,” Journal of Lasers in Medical Sciences 13 (2022): e31. 36743152 10.34172/jlms.2022.31 PMC9841374

[12] A. C. Tricco , E. Lillie , W. Zarin , et al., “PRISMA Extension for Scoping Reviews (PRISMA‐ScR): Checklist and Explanation,” Annals of Internal Medicine 169, no. 7 (2018): 467–473. 30178033 10.7326/M18-0850

[13] L. S. Prott , A. Carrasco‐Labra , P. C. Gierthmuehlen , and M. B. Blatz , “How to Conduct and Publish Systematic Reviews and Meta‐Analyses in Dentistry,” Journal of Esthetic and Restorative Dentistry 37, no. 1 (2025): 14–27. 39535363 10.1111/jerd.13366 PMC11913207

[14] M. Kimmich and C. F. Stappert , “Intraoral Treatment of Veneering Porcelain Chipping of Fixed Dental Restorations: A Review and Clinical Application,” Journal of the American Dental Association 144, no. 1 (2013): 31–44. 23283924 10.14219/jada.archive.2013.0011

[15] P. Kanzow , A. Wiegand , F. Schwendicke , and G. Göstemeyer , “Same, Same, but Different? A Systematic Review of Protocols for Restoration Repair,” Journal of Dentistry 86 (2019): 1–16. 31108118 10.1016/j.jdent.2019.05.021

[16] J. P. Matinlinna and P. K. Vallittu , “Bonding of Resin Composites to Etchable Ceramic Surfaces—An Insight Review of the Chemical Aspects on Surface Conditioning,” Journal of Oral Rehabilitation 34, no. 8 (2007): 622–630. 17650173 10.1111/j.1365-2842.2005.01569.x

[17] R. Li , Y. C. Sun , C. Wang , and P. Gao , “Bonding of an Opaque Resin to Silane‐Treated Porcelain,” Bio‐Medical Materials and Engineering 24, no. 6 (2014): 2117–2125. 25226909 10.3233/BME-141022

[18] B. K. Kim , H. E. Bae , J. S. Shim , and K. W. Lee , “The Influence of Ceramic Surface Treatments on the Tensile Bond Strength of Composite Resin to All‐Ceramic Coping Materials,” Journal of Prosthetic Dentistry 94, no. 4 (2005): 357–362. 16198173 10.1016/j.prosdent.2005.08.012

[19] F. G. Panah , S. M. Rezai , and L. Ahmadian , “The Influence of Ceramic Surface Treatments on the Micro‐Shear Bond Strength of Composite Resin to IPS Empress 2,” Journal of Prosthodontics 17, no. 5 (2008): 409–414. 18717831 10.1111/j.1532-849X.2007.00296.x

[20] M. Kazak , M. G. Subasi , D. O. Ozdas , S. Zorlu , A. Cilingir , and S. Gunal , “The Effects of Nutritional Habits on Leucite‐Based Ceramic Repaired With Nanohybrid Composites,” Journal of Adhesion Science and Technology 33, no. 15 (2019): 1705–1714.

[21] M. Duzyol , O. Sagsoz , N. Polat Sagsoz , N. Akgul , and M. Yildiz , “The Effect of Surface Treatments on the Bond Strength Between CAD/CAM Blocks and Composite Resin,” Journal of Prosthodontics 25, no. 6 (2016): 466–471. 26216441 10.1111/jopr.12322

[22] İ. H. Uzun , M. A. Malkoç , N. T. Polat , and A. T. Öğreten , “The Effect of Repair Protocols on Shear Bond Strength to Zirconia and Veneering Porcelain,” Journal of Adhesion Science and Technology 30, no. 16 (2016): 1741–1753.

[23] K. Barutcigil and O. Kirmali , “The Effect of Different Surface Treatments on Repair With Composite Resin of Ceramic,” Nigerian Journal of Clinical Practice 23, no. 3 (2020): 355–361. 32134035 10.4103/njcp.njcp_409_19

[24] B. Kukiattrakoon and K. Thammasitboon , “The Effect of Different Etching Times of Acidulated Phosphate Fluoride Gel on the Shear Bond Strength of High‐Leucite Ceramics Bonded to Composite Resin,” Journal of Prosthetic Dentistry 98, no. 1 (2007): 17–23. 17631170 10.1016/S0022-3913(07)60033-X

[25] N. Ueda , T. Takagaki , T. Nikaido , R. Takahashi , M. Ikeda , and J. Tagami , “The Effect of Different Ceramic Surface Treatments on the Repair Bond Strength of Resin Composite to Lithium Disilicate Ceramic,” Dental Materials Journal 40, no. 5 (2021): 1073–1079. 33883329 10.4012/dmj.2020-362

[26] H. Özdemir and N. D. Yanikoglu , “The Bond Strength of Nanohybrid and Nanoceramic Composites to Feldspathic Porcelain,” Contemporary Clinical Dentistry 8, no. 4 (2017): 558–564. 29326506 10.4103/ccd.ccd_504_17 PMC5754976

[27] C. A. Neis , N. L. Albuquerque , S. Albuquerque Ide , et al., “Surface Treatments for Repair of Feldspathic, Leucite‐ and Lithium Disilicate‐Reinforced Glass Ceramics Using Composite Resin,” Brazilian Dental Journal 26 (2015): 152–155. 25831106 10.1590/0103-6440201302447

[28] E. Moravej‐Salehi , E. Moravej‐Salehi , and A. Valian , “Surface Topography and Bond Strengths of Feldspathic Porcelain Prepared Using Various Sandblasting Pressures,” Journal of Investigative and Clinical Dentistry 7, no. 4 (2016): 347–354. 26088205 10.1111/jicd.12171

[29] N. Mohammadi , M. S. Shahabi , S. Kimyai , F. P. Azar , and M. E. E. Chaharom , “Shear Bond Strengths of Methacrylate‐ and Silorane‐Based Composite Resins to Feldspathic Porcelain Using Different Adhesive Systems,” Journal of Dental Research, Dental Clinics, Dental Prospects 9, no. 3 (2015): 181–187. 26697151 10.15171/joddd.2015.033 PMC4682015

[30] T. Külünk and Y. Şinasi Saraç , “Surface Roughness, Wettability and Bond Strength of Three Different Dental Repair Systems,” Materials Research Innovations 15, no. 1 (2011): 17–23.

[31] H. Kilinc , F. A. Sanal , and S. Turgut , “Shear Bond Strengths of Aged and Non‐Aged CAD/CAM Materials After Different Surface Treatments,” Journal of Advanced Prosthodontics 12, no. 5 (2020): 273–282. 33149848 10.4047/jap.2020.12.5.273 PMC7604239

[32] H. Pedrazzi , C. Y. Takeuchi , S. S. Cioffi , M. R. Galvão , M. F. de Andrade , and O. L. Bezzon , “Shear Bond Strength of Repairs in Porcelain Conditioned With Laser,” Microscopy Research and Technique 75, no. 12 (2012): 1639–1645. 22851485 10.1002/jemt.22109

[33] Ö. Üstün , I. K. Büyükhatipoğlu , and A. Seçilmiş , “Shear Bond Strength of Repair Systems to New CAD/CAM Restorative Materials,” Journal of Prosthodontics 27, no. 8 (2018): 748–754. 27880011 10.1111/jopr.12564

[34] M. S. Akyil , A. Yilmaz , O. F. Karaalioğlu , and Z. Y. Duymuş , “Shear Bond Strength of Repair Composite Resin to an Acid‐Etched and a Laser‐Irradiated Feldspathic Ceramic Surface,” Photomedicine and Laser Surgery 28, no. 4 (2010): 539–545. 19852588 10.1089/pho.2009.2586

[35] A. A. Baiomy , J. F. Younis , and A. H. Khalil , “Shear Bond Strength of Composite Repair System to Bilayered Zirconia Using Different Surface Treatments (In Vitro Study),” Brazilian Dental Science 23, no. 1 (2020): 11.

[36] R. M. Falah and Z. M. Abdul Ameer , “Shear Bond Strength of Aged CAD/CAM Ceramic Materials Repaired by Resin Composite In Vitro Using Different Repair Adhesives,” Journal of Research in Medical and Dental Science 8, no. 1 (2020): 61–67.

[37] U. Erdemir , H. S. Sancakli , E. Sancakli , et al., “Shear Bond Strength of a New Self‐Adhering Flowable Composite Resin for Lithium Disilicate‐Reinforced CAD/CAM Ceramic Material,” Journal of Advanced Prosthodontics 6, no. 6 (2014): 434–443. 25551002 10.4047/jap.2014.6.6.434 PMC4279040

[38] N. B. Hassan , B. M. A. Hussein , and A. A. Mohammed , “Role of CO_2_ Laser on SBS Between Dental Porcelain and Composite Resin Repair Process,” International Journal of Dentistry 2023 (2023): 1427183.

[39] M. H. Hwang , H. R. Kim , and T. Y. Kwon , “Repairing Fractured Ceramic Veneer With CAD/CAM Ceramic Blocks: A Preliminary Tensile Bond Strength Study,” Materials and Technologies 34, no. 1 (2019): 43–50.

[40] A. M. Maawadh , T. Almohareb , R. S. Al‐Hamdan , et al., “Repair Strength and Surface Topography of Lithium Disilicate and Hybrid Resin Ceramics With LLLT and Photodynamic Therapy in Comparison to Hydrofluoric Acid,” Journal of Applied Biomaterials & Functional Materials 18 (2020): 2280800020966938. 33270475 10.1177/2280800020966938

[41] M. Karci , N. Demir , M. Subasi , and M. Gokkaya , “Shear Bond Strength of a Novel Porcelain Repair System for Different Computer‐Aided Design/Computer‐Assisted Manufacturing Ceramic Materials,” Nigerian Journal of Clinical Practice 21, no. 4 (2018): 507–513. 29607866 10.4103/njcp.njcp_127_17

[42] S. Flury , F. A. Dulla , and A. Peutzfeldt , “Repair Bond Strength of Resin Composite to Restorative Materials After Short‐ and Long‐Term Storage,” Dental Materials 35, no. 9 (2019): 1205–1213. 31146960 10.1016/j.dental.2019.05.008

[43] A. S. Ataol and G. Ergun , “Repair Bond Strength of Resin Composite to Bilayer Dental Ceramics,” Journal of Advanced Prosthodontics 10, no. 2 (2018): 101–112. 29713430 10.4047/jap.2018.10.2.101 PMC5917101

[44] S. Çınar and Ö. Kırmalı , “Repair Bond Strength of Composite Resin to Zirconia Restorations After Different Thermal Cycles,” Journal of Advanced Prosthodontics 11, no. 5 (2019): 297–304. 31754420 10.4047/jap.2019.11.5.297 PMC6856308

[45] A. Kameyama , A. Haruyama , A. Tanaka , et al., “Repair Bond Strength of a Resin Composite to Plasma‐Treated or UV‐Irradiated CAD/CAM Ceramic Surface,” Coatings 8, no. 7 (2018): 230.

[46] Y. Bayraktar , M. Arslan , and Z. Demirtag , “Repair Bond Strength and Surface Topography of Resin‐Ceramic and Ceramic Restorative Blocks Treated by Laser and Conventional Surface Treatments,” Microscopy Research and Technique 84, no. 6 (2021): 1145–1154. 33615613 10.1002/jemt.23672

[47] R. M. D. Melo , L. F. Valandro , and M. A. Bottino , “Microtensile Bond Strength of a Repair Composite to Leucite‐Reinforced Feldspathic Ceramic,” Brazilian Dental Journal 18, no. 4 (2007): 314–319. 18278302 10.1590/s0103-64402007000400008

[48] M. Ghavam , M. Soleimanpour , S. S. Hashemikamangar , H. Ebrahimi , and M. J. Kharazifard , “Microshear Bond Strength of Self‐Adhesive Composite to Ceramic After Mechanical, Chemical and Laser Surface Treatments,” Laser Therapy 26, no. 4 (2017): 297–304. 29434430 10.5978/islsm.17-OR-19 PMC5801455

[49] S. H. Kim , Y. K. Lee , B. S. Lim , S. H. Rhee , and H. C. Yang , “Metameric Effect Between Dental Porcelain and Porcelain Repairing Resin Composite,” Dental Materials 23, no. 3 (2007): 374–379. 16540161 10.1016/j.dental.2006.01.027

[50] E. Tokar , S. Polat , and C. Ozturk , “Repair Bond Strength of Composite to Er,Cr:YSGG Laser Irradiated Zirconia and Porcelain Surfaces,” Biomedical Journal 42, no. 3 (2019): 193–199. 31466713 10.1016/j.bj.2019.02.001 PMC6717752

[51] D. A. Suárez‐Moya , A. C. Cruz‐González , and J. N. Calvo‐Ramírez , “Interaction of a Universal Adhesive With Different Surface Treatments With Feldespathic Ceramics,” Saudi Dental Journal 31, no. 3 (2019): 350–354. 31337939 10.1016/j.sdentj.2019.03.008 PMC6626276

[52] R. Turunç‐Oğuzman and S. Şişmanoğlu , “Influence of Surface Treatments and Adhesive Protocols on Repair Bond Strength of Glass‐Matrix and Resin‐Matrix CAD/CAM Ceramics,” Journal of Esthetic and Restorative Dentistry 35, no. 8 (2023): 1322–1331. 37680089 10.1111/jerd.13131

[53] A. Attia , “Influence of Surface Treatment and Cyclic Loading on the Durability of Repaired All‐Ceramic Crowns,” Journal of Applied Oral Science 18, no. 2 (2010): 194–200. 20485932 10.1590/S1678-77572010000200015 PMC5349757

[54] R. Al‐Thagafi , W. Al‐Zordk , and S. Saker , “Influence of Surface Conditioning Protocols on Reparability of CAD/CAM Zirconia‐Reinforced Lithium Silicate Ceramic,” Journal of Adhesive Dentistry 18, no. 2 (2016): 135–141. 27042707 10.3290/j.jad.a35909

[55] J. R. Queiroz , R. O. Souza , L. Nogueira Junior, Jr. , M. Ozcan , and M. A. Bottino , “Influence of Acid‐Etching and Ceramic Primers on the Repair of a Glass Ceramic,” General Dentistry 60, no. 2 (2012): e79–e85. 22414522

[56] H. Kocaağaoğlu , T. Manav , and H. Albayrak , “In Vitro Comparison of the Bond Strength Between Ceramic Repair Systems and Ceramic Materials and Evaluation of the Wettability,” Journal of Prosthodontics 26, no. 3 (2017): 238–243. 26524614 10.1111/jopr.12381

[57] P. F. J. S. da Cunha , J. G. Tavares , A. M. Spohr , M. C. Bellan , C. H. Bueno , and L. I. Cardoso , “Examining the Effects of Acid Etching Duration on the Bond Strength Between Two CAD/CAM Materials and One Composite Resin,” Odontology 110, no. 1 (2022): 113–119. 34363147 10.1007/s10266-021-00644-x

[58] B. Karabulut Gençer , E. Acar , and B. Tarçın , “Evaluation of Shade Matching in the Repair of Indirect Restorative Materials With Universal Shade Composites,” European Oral Research 57, no. 1 (2023): 41–48. 37020638 10.26650/eor.20231076495 PMC10069798

[59] S. Polat , E. Tokar , N. V. Asar , and O. Kirmali , “Evaluation of Efficacy of Various Surface Conditioning Methods on the Repair Bond Strength of Composite to Different Fracture Types of Zirconia Ceramics,” Scanning 2021 (2021): 5537761. 34131464 10.1155/2021/5537761 PMC8178005

[60] A. U. Guler , F. Yilmaz , C. Ural , and E. Guler , “Evaluation of 24‐Hour Shear Bond Strength of Resin Composite to Porcelain According to Surface Treatment,” Journal of Prosthetic Dentistry 94, no. 6 (2005): 538. 15889665

[61] F. Sanal and H. Kilinc , “Evaluating Ceramic Repair Materials in Terms of Bond Strength and Color Stability,” International Journal of Prosthodontics 33, no. 5 (2020): 536–545. 32956435 10.11607/ijp.6760

[62] A. S. Ataol and G. Ergun , “Effects of Surface Treatments on Repair Bond Strength of a New CAD/CAM ZLS Glass Ceramic and Two Different Types of CAD/CAM Ceramics,” Journal of Oral Science 60, no. 2 (2018): 201–211. 29925704 10.2334/josnusd.17-0109

[63] F. F. Mohamed , M. Finkelman , R. Zandparsa , H. Hirayama , and G. Kugel , “Effects of Surface Treatments and Cement Types on the Bond Strength of Porcelain‐to‐Porcelain Repair,” Journal of Prosthodontics 23, no. 8 (2014): 618–625. 25066092 10.1111/jopr.12194

[64] V. Sattabanasuk , P. Charnchairerk , L. Punsukumtana , and M. F. Burrow , “Effects of Mechanical and Chemical Surface Treatments on the Resin‐Glass Ceramic Adhesion Properties,” Journal of Investigative and Clinical Dentistry 8, no. 3 (2017): e12220. 10.1111/jicd.12220 27282642

[65] B. Unalan Degirmenci , A. Degirmenci , and B. Karadag Naldemir , “Effects of Er,Cr:YSGG Laser on Repair Bond Strength of 5‐Year Water‐Aged and Non‐Aged CAD/CAM Ceramics,” International Journal of Applied Ceramic Technology 19, no. 3 (2022): 1594–1604.

[66] B. R. Huang , X. Y. Wang , and X. J. Gao , “Effects of Different Surface Treatments on Ceramic Repairs With Composite,” Chinese Journal of Dental Research 16, no. 2 (2013): 111–117. 24436946

[67] D. Saraç , Y. S. Saraç , S. Külünk , and A. Erkoçak , “Effect of Various Surface Treatments on the Bond Strength of Porcelain Repair,” International Journal of Periodontics & Restorative Dentistry 33, no. 4 (2013): e120–e126. 23820715 10.11607/prd.1362

[68] N. Kiomarsi , A. Jarrah , N. Chiniforoush , S. Hashemikamangar , and M. Kharazifard , “Effect of Surface Treatment With Laser on Repair Bond Strength of Composite Resin to Ceramic,” Dental Research Journal 19, no. 1 (2022): 30. 35432793 PMC9006155

[69] R. C. R. Colares , J. R. Neri , A. M. B. D. Souza , K. M. D. F. Pontes , J. S. Mendonca , and S. L. Santiago , “Effect of Surface Pretreatments on the Microtensile Bond Strength of Lithium‐Disilicate Ceramic Repaired With Composite Resin,” Brazilian Dental Journal 24, no. 4 (2013): 349–352. 24173254 10.1590/0103-6440201301960

[70] S. Şişmanoğlu , A. T. Gürcan , Z. Yıldırım‐Bilmez , R. Turunç‐Oğuzman , and B. Gümüştaş , “Effect of Surface Treatments and Universal Adhesive Application on the Microshear Bond Strength of CAD/CAM Materials,” Journal of Advanced Prosthodontics 12, no. 1 (2020): 22–32. 32128083 10.4047/jap.2020.12.1.22 PMC7040451

[71] S. M. R. Hakimaneh , S. S. Shayegh , M. Ghavami‐Lahiji , A. Chokr , and A. Moraditalab , “Effect of Silane Heat Treatment by Laser on the Bond Strength of a Repair Composite to Feldspathic Porcelain,” Journal of Prosthodontics 29, no. 1 (2020): 49–55. 29380487 10.1111/jopr.12762

[72] B. Yildirim , D. Recen , and G. Paken , “Effect of Self‐Etching Ceramic Primer on the Bond Strength of Feldspathic Porcelain Repair,” Journal of Adhesion Science and Technology 33, no. 14 (2019): 1598–1610.

[73] W.‐S. Oh and C. Shen , “Effect of Flame Cleaning of Ceramic Surface on the Bond Strength of Composite to Ceramic,” Journal of Oral Rehabilitation 32, no. 2 (2005): 141–144. 15641981 10.1111/j.1365-2842.2004.01398.x

[74] A. Valian , E. Moravej Salehi , M. Mahmoudzadeh , and N. Kheirkhah Dabagh , “Effect of Different Surface Treatment on the Repair Bond Strength of Feldspathic Porcelain,” Dental and Medical Problems 58, no. 1 (2021): 107–113. 33847469 10.17219/dmp/130101

[75] H. Zaghloul , D. W. Elkassas , and M. F. Haridy , “Effect of Incorporation of Silane in the Bonding Agent on the Repair Potential of Machinable Esthetic Blocks,” European Journal of Dentistry 8, no. 1 (2014): 44–52. 24966745 10.4103/1305-7456.126240 PMC4054031

[76] A. U. Güler , I. B. Sarikaya , E. Güler , and A. Yücel , “Effect of Filler Ratio in Adhesive Systems on the Shear Bond Strength of Resin Composite to Porcelains,” Operative Dentistry 34, no. 3 (2009): 299–305. 19544819 10.2341/08-88

[77] M. Atala and E. Yeğin , “Effect of Different Universal Bonding Agent Procedures on Repair of Feldspathic and Hybrid Ceramics,” International Journal of Prosthodontics 35, no. 3 (2022): 330–337. 35727264 10.11607/ijp.7753

[78] M. Tavares , F. Lb Amaral , R. Y. Basting , C. P. Turssi , and F. Mg França , “Effect of Different Design and Surface Treatment on the Load‐to‐Failure of Ceramic Repaired With Composite,” Acta Odontológica Latinoamericana 37, no. 1 (2024): 88–95. 38920130 10.54589/aol.37/1/88 PMC11212215

[79] P. M. Soares , A. M. de Oliveira Dal Piva , G. K. R. Pereira , et al., “Effect of Brushing Simulation on the Wear Behavior of Repaired CAD‐CAM Restorations,” International Dental Journal 74, no. 5 (2024): 999–1005. 38461097 10.1016/j.identj.2024.02.012 PMC11561493

[80] B. Tarcin , G. Sinmazisik , F. Ozer , and T. Gülmez , “Effect of Different Surface Applications and Adhesive Systems on Bond Strength of Porcelain Repair Material,” Key Engineering Materials 493–494 (2011): 643–648.

[81] P. H. Corazza , R. F. de Carvalho , R. O. de Assunção e Souza , M. A. Bottino , and M. Özcan , “Effect of Aging Type and Aged Unit on the Repair Strength of Resin Composite to Feldspathic Porcelain in Testing Microtensile Bond Strength,” Journal of Adhesion Science and Technology 30, no. 4 (2016): 434–442.

[82] A. U. Güler , F. Yilmaz , M. Yenisey , E. Güler , and C. Ural , “Effect of Acid Etching Time and a Self‐Etching Adhesive on the Shear Bond Strength of Composite Resin to Porcelain,” Journal of Adhesive Dentistry 8, no. 1 (2006): 21–25. 16536340

[83] M. Carrabba , A. Vichi , C. Louca , and M. Ferrari , “Comparison of Traditional and Simplified Methods for Repairing CAD/CAM Feldspathic Ceramics,” Journal of Advanced Prosthodontics 9, no. 4 (2017): 257–264. 28874992 10.4047/jap.2017.9.4.257 PMC5582091

[84] R. Schellenberg and M. Özcan , “Comparison of Repair Protocols for Veneered Zirconia as a Function of Surface Conditioning Parameters, Ceramic Primer Types and Defect Sizes,” Journal of Adhesion Science and Technology 35, no. 19 (2021): 2110–2123.

[85] P. K. Lundvall , E. Ruyter , H. J. Rønold , and K. Ekstrand , “Comparison of Different Etching Agents and Repair Materials Used on Feldspathic Porcelain,” Journal of Adhesion Science and Technology 23, no. 7–8 (2009): 1177–1186.

[86] H. Kumchai , P. Juntavee , A. F. Sun , and D. Nathanson , “Comparing the Repair of Veneered Zirconia Crowns With Ceramic or Composite Resin: An In Vitro Study,” Dentistry Journal 8, no. 2 (2020): 37. 32349281 10.3390/dj8020037 PMC7345289

[87] Y. K. Lee , “Changes in the Translucency of Porcelain and Repairing Resin Composite by the Illumination,” Dental Materials 23, no. 4 (2007): 492–497. 16631922 10.1016/j.dental.2006.03.004

[88] J. R. Chen , K. Oka , T. Kawano , T. Goto , and T. Ichikawa , “Carbon Dioxide Laser Application Enhances the Effect of Silane Primer on the Shear Bond Strength Between Porcelain and Composite Resin,” Dental Materials Journal 29, no. 6 (2010): 731–737. 21099155 10.4012/dmj.2009-106

[89] M. AlRabiah , N. Labban , J. A. Levon , et al., “Bond Strength and Durability of Universal Adhesive Agents With Lithium Disilicate Ceramics: A Shear Bond Strength Study,” Journal of Adhesion Science and Technology 32, no. 6 (2018): 580–589.

[90] L. Al Deeb , “Application of Low‐Level Laser Therapy in Veneer Repair of Lithium Disilicate and Y‐TZP Restorations,” Bioscience Biotechnology Research Communications 13, no. 1 (2020): 68–72.

[91] I. R. Blum , N. Nikolinakos , C. D. Lynch , N. H. F. Wilson , B. J. Millar , and D. C. Jagger , “An In Vitro Comparison of Four Intra‐Oral Ceramic Repair Systems,” Journal of Dentistry 40, no. 11 (2012): 906–912. 22819811 10.1016/j.jdent.2012.07.008

[92] R. A. Al‐Askary , W. M. O. Al‐Ashou , and S. N. Hassoon , “Repair Bond Strength of Composite Resin to Dental Ceramic Using Various Surface Treatments: An In Vitro Study,” Journal of International Society of Preventive and Community Dentistry 14, no. 5 (2024): 388–395. 39677524 10.4103/jispcd.jispcd_71_24 PMC11637163

[93] N. Naderi , S. Majidinia , M. J. Moghaddas , Z. Shooshtari , and M. Hoseinzadeh , “Surface Modification Effect on the Repair Bond Strength of Hybrid and Non‐Hybrid Ceramics,” BMC Oral Health 25, no. 1 (2025): 1131. 40635017 10.1186/s12903-025-06527-9 PMC12239320

[94] M. S. de Bessa , L. C. N. Marinho , L. M. de Miranda , et al., “Repair Bond Strength of Resin Composite to CAD/CAM Glass‐Ceramic: Influence of Cleaning Methods, Surface Treatments, and Aging,” Journal of Dentistry 154 (2025): 105568. 39805494 10.1016/j.jdent.2025.105568

[95] M. A. Sagen , B. Eriksen , K. Moland , and B. E. Dahl , “An In Vitro Study on the Bond Strength Between Composite Resin and Porcelain Using Different Surface Treatment and Bonding Methods,” European Journal of Oral Sciences 133, no. 3 (2025): e70014. 40325494 10.1111/eos.70014 PMC12092814

[96] S. Al‐Hmadi , F. Erol , and M. G. Çelik , “Shear Bond Strengths of Five Porcelain Repair Systems to Zirconia Infrastructures,” European Oral Research 56, no. 2 (2022): 55–60. 36003840 10.26650/eor.2022962372 PMC9377777

[97] S. R. Habib , S. Bajunaid , A. Almansour , et al., “Shear Bond Strength of Veneered Zirconia Repaired Using Various Methods and Adhesive Systems: A Comparative Study,” Polymers 13, no. 6 (2021): 910. 33809539 10.3390/polym13060910 PMC7998840

[98] P. M. Soares , L. S. da Rosa , R. O. Pilecco , et al., “Repair of Monolithic Zirconia Restorations With Different Direct Resin Composites: Effect on the Fatigue Bonding and Mechanical Performance,” Clinical Oral Investigations 28, no. 2 (2024): 149. 38355823 10.1007/s00784-024-05542-4 PMC10866771

[99] O. Kirmali , Ç. Barutcigil , M. M. Ozarslan , K. Barutcigil , and O. T. Harorlı , “Repair Bond Strength of Composite Resin to Sandblasted and Laser Irradiated Y‐TZP Ceramic Surfaces,” Scanning 37, no. 3 (2015): 186–192. 25715193 10.1002/sca.21197

[100] A. A. Gouda , A. D. Abogabal , R. Tammam , and A. Sleem , “Investigation the Function of Surface Conditioning Parameters and Different Adhesive Resin Types for the Chairside Repair Protocols of Translucent Zirconia (5y) Dental Restorations,” Journal of Pharmaceutical Negative Results 14 (2023): 785–795.

[101] H. Mahgoli , M. Arshad , K. Rasouli , A. A. Sobati , and A. R. Shamshiri , “Repair Bond Strength of Composite to Zirconia Ceramic Using Two Types of Zirconia Primers,” Frontiers in Dentistry 16, no. 5 (2019): 342–350. 32123874 10.18502/fid.v16i5.2279 PMC7040558

[102] B. Seabra , S. Arantes‐Oliveira , and J. Portugal , “Influence of Multimode Universal Adhesives and Zirconia Primer Application Techniques on Zirconia Repair,” Journal of Prosthetic Dentistry 112, no. 2 (2014): 182–187. 24445031 10.1016/j.prosdent.2013.10.008

[103] R. Li , C. Wang , S. Q. Ma , et al., “High Bonding Strength Between Zirconia and Composite Resin Based on Combined Surface Treatment for Dental Restorations,” Journal of Applied Biomaterials & Functional Materials 18 (2020): 2280800020928655. 33147097 10.1177/2280800020928655

[104] W. Libecki , A. Elsayed , F. Lehmann , and M. Kern , “Efficacy of Different Surface Treatments for Intraoral Repair of Veneered Zirconia Frameworks,” Journal of Adhesive Dentistry 19, no. 4 (2017): 323–329. 28849798 10.3290/j.jad.a38891

[105] F. Shafiei , Z. Fattah , N. Kiomarsi , and M. H. Dashti , “Influence of Primers and Additional Resin Layer on Zirconia Repair Bond Strength,” Journal of Prosthodontics 28, no. 7 (2019): 826–832. 30582263 10.1111/jopr.13011

[106] I. H. Han , D. W. Kang , C. H. Chung , H. C. Choe , and M. K. Son , “Effect of Various Intraoral Repair Systems on the Shear Bond Strength of Composite Resin to Zirconia,” Journal of Advanced Prosthodontics 5, no. 3 (2013): 248–255. 24049565 10.4047/jap.2013.5.3.248 PMC3774938

[107] B. R. G. Ribeiro , M. R. G. R. Caldas , A. A. Almeida, Jr. , R. G. Fonseca , and G. L. Adabo , “Effect of Surface Treatments on Repair With Composite Resin of a Partially Monoclinic Phase Transformed Yttrium‐Stabilized Tetragonal Zirconia,” Journal of Prosthetic Dentistry 119, no. 2 (2018): 286–291. 28533011 10.1016/j.prosdent.2017.02.014

[108] T. Alnassar , “Effect of Surface Treatments and Aging Solutions on the Repair Bond Strength of Composite Resin to Zirconia,” Journal of Biomaterials and Tissue Engineering 7, no. 10 (2017): 1045–1050.

[109] A. Elraggal , X. Chen , and N. Silikas , “Effect of Sandblasting With Fluorapatite Glass‐Ceramic Powder and Chemical Primers/Adhesives on Shear Bond Strength of Indirect Repairing Composite to Zirconia,” Operative Dentistry 47, no. 5 (2022): 574–584. 36121727 10.2341/21-108-L

[110] A. Elraggal and N. Silikas , “Effect of Air‐Abraded Versus Laser‐Fused Fluorapatite Glass‐Ceramics on Shear Bond Strength of Repair Materials to Zirconia,” Materials 14, no. 6 (2021): 1468. 33802778 10.3390/ma14061468 PMC8002453

[111] P. M. Soares , L. S. da Rosa , R. O. Pilecco , et al., “Cyclic Fatigue of a Repaired 4YSZ Ceramic: Effect of the Repair Protocol on the Adhesive and Mechanical Behavior,” Heliyon 10, no. 1 (2024): e23709. 38187296 10.1016/j.heliyon.2023.e23709 PMC10767202

[112] P. Cristoforides , R. Amaral , L. G. May , M. A. Bottino , and L. F. Valandro , “Composite Resin to Yttria Stabilized Tetragonal Zirconia Polycrystal Bonding: Comparison of Repair Methods,” Operative Dentistry 37, no. 3 (2012): 263–271. 22313269 10.2341/11-193-L

[113] K. Fathpour , M. Nili Ahmadabadi , R. Atash , and A. H. Fathi , “Effect of Different Surface Treatment Methods on the Shear Bond Strength of Resin Composite/Zirconia for Intra‐Oral Repair of Zirconia Restorations,” European Journal of Dentistry 17, no. 3 (2023): 809–817. 36220116 10.1055/s-0042-1756475 PMC10569880

[114] N. Barchetta , A. Silva , N. Domingues , et al., “Cleaning and Surface Treatment Protocols for Repair of Aged Y‐TZP With Composite Resin,” International Journal of Periodontics & Restorative Dentistry 41, no. 1 (2021): e19–e26. 33528455 10.11607/prd.4915

[115] G. Barragan , F. Chasqueira , S. Arantes‐Oliveira , and J. Portugal , “Ceramic Repair: Influence of Chemical and Mechanical Surface Conditioning on Adhesion to Zirconia,” Oral Health and Dental Management 13, no. 2 (2014): 155–158. 24984615

[116] R. A. Dos Santos , E. A. de Lima , L. S. Mendonça , et al., “Can Universal Adhesive Systems Bond to Zirconia?,” Journal of Esthetic and Restorative Dentistry 31, no. 6 (2019): 589–594. 31456314 10.1111/jerd.12521

[117] S. J. Lee , C. W. Cheong , R. F. Wright , and B. M. Chang , “Bond Strength of the Porcelain Repair System to All‐Ceramic Copings and Porcelain,” Journal of Prosthodontics 23, no. 2 (2014): 112–116. 23725343 10.1111/jopr.12064

[118] M. Janson , V. Bassier , A. Liebermann , C. M. Schoppmeier , and M. Di Gregorio‐Schininà , “Composite Repair on Zirconia: Influence of Different Sandblasting Pretreatments and Various Universal Adhesives on Shear Bond Strength,” Journal of Adhesive Dentistry 27, no. 1 (2025): 53–64. 40237152 10.3290/j.jad.c_1988 PMC12020428

[119] T. S. Goia , F. P. Leite , L. F. Valandro , M. Ozcan , and M. A. Bottino , “Repair Bond Strength of a Resin Composite to Alumina‐Reinforced Feldspathic Ceramic,” International Journal of Prosthodontics 19, no. 4 (2006): 400–402. 16900826

[120] B. Höller , R. Belli , A. Petschelt , U. Lohbauer , and J. I. Zorzin , “Influence of Simulated Oral Conditions on Different Pretreatment Methods for the Repair of Glass‐Ceramic Restorations,” Journal of Adhesive Dentistry 24 (2022): 57–66. 35227047 10.3290/j.jad.b2701717 PMC11734243

[121] M. Sadeghi , A. Davari , A. Abolghasami Mahani , and H. Hakimi , “Influence of Different Power Outputs of Er:YAG Laser on Shear Bond Strength of a Resin Composite to Feldspathic Porcelain,” Journal of Dentistry, Shiraz University of Medical Sciences 16, no. 1 (2015): 30–36. PMC4345111 25759855

[122] O. Kirmali , A. Kapdan , O. T. Harorli , C. Barutcugil , and M. M. Ozarslan , “Efficacy of Ceramic Repair Material on the Bond Strength of Composite Resin to Zirconia Ceramic,” Acta Odontologica Scandinavica 73, no. 1 (2015): 28–32. 25373516 10.3109/00016357.2014.946963

[123] M. Benli , E. H. H. Kilic , B. E. Gumus , and I. Turkyilmaz , “Effects of Various Laser Treatments on Surface Characterization and Repair Bond Strength of Zirconia‐Reinforced Lithium Silicate Ceramics,” International Journal of Prosthodontics 37, no. 1 (2024): 92–94. 35323828 10.11607/ijp.7951

[124] Z. A. Al Jeaidi , “Influence of Resin Removal Treatments on the Surface Topography and Strength of De‐Bonded Lithium Disilicate Ceramic,” Journal of Applied Biomaterials & Functional Materials 20 (2022): 2280800020944015. 35277088 10.1177/2280800020944015

[125] M. E. Ebrahimi Chaharom , F. Pournaghi Azar , N. Mohammadi , and R. Nasiri , “Effect of Surface Preparation With Nd:YAG and Er,Cr:YSGG Lasers on the Repair Bond Strength of Lithium Disilicate Glass Ceramic to a Silorane‐Based Composite Resin,” Journal of Dental Research Dental Clinics Dental Prospects 12, no. 1 (2018): 12–17. 29732016 10.15171/joddd.2018.003 PMC5928469

[126] A. H. Veríssimo , D. M. Duarte Moura , A. M. de Oliveira Dal Piva , et al., “Effect of Different Repair Methods on the Bond Strength of Resin Composite to CAD/CAM Materials and Microorganisms Adhesion: An In Situ Study,” Journal of Dentistry 93 (2020): 103266. 31863809 10.1016/j.jdent.2019.103266

[127] M. Özcan , L. F. Valandro , R. Amaral , F. Leite , and M. A. Bottino , “Bond Strength Durability of a Resin Composite on a Reinforced Ceramic Using Various Repair Systems,” Dental Materials 25, no. 12 (2009): 1477–1483. 19671476 10.1016/j.dental.2009.06.020

[128] R. Meshramkar and S. Sajjan , “A Comparative Evaluation of Shear Bond Strength of Porcelain and Composite Using Different Bonding Agents—An In Vitro Study,” Journal of Indian Prosthodontic Society 10, no. 1 (2010): 36–40. 23204719 10.1007/s13191-010-0003-3 PMC3453181

[129] C. D. Bergoli , R. F. de Carvalho , J. N. Luz , M. S. Luz , D. K. Meincke , and G. S. Saavedra , “Ceramic Repair Without Hydrofluoric Acid,” Journal of Adhesive Dentistry 18, no. 4 (2016): 283–287. 27222888 10.3290/j.jad.a36152

[130] B. K. Gençer , A. A. Şenol , E. Acar , P. Y. Atalı , B. Tarçın , and M. Özcan , “Effect of Surface Conditioning Protocols on the Repair Bond Strength of Resin Composite to CAD/CAM Blocks: Bioactive‐Glass, Silica‐Coated Alumina, or Aluminum Oxide?,” European Journal of Oral Sciences 133, no. 4 (2025): e70016. 40358426 10.1111/eos.70016 PMC12269539

[131] E. Çağlayan , R. Sasany , B. N. Bolat , et al., “Optical Properties of Repaired Additively Manufactured Resin Composites and Zirconia and Subtractively Manufactured Ceramics: A Comparative Study of Composite Resins,” BMC Oral Health 25, no. 1 (2025): 144. 39871238 10.1186/s12903-025-05538-w PMC11773952

[132] R. I. Falacho , E. A. Melo , J. A. Marques , J. C. Ramos , F. Guerra , and M. B. Blatz , “Clinical In‐Situ Evaluation of the Effect of Rubber Dam Isolation on Bond Strength to Enamel,” Journal of Esthetic and Restorative Dentistry 35, no. 1 (2023): 48–55. 36325593 10.1111/jerd.12979

[133] G. Almeida , J. A. Marques , B. van Meerbeek , J. C. Ramos , and R. I. Falacho , “Particle Abrasion as a Pre‐Bonding Dentin Surface Treatment: A Scoping Review,” Journal of Esthetic and Restorative Dentistry 36, no. 5 (2024): 746–760. 38130045 10.1111/jerd.13183

